# The Role of Natural Polyphenols in the Prevention and Treatment of Cervical Cancer—An Overview

**DOI:** 10.3390/molecules21081055

**Published:** 2016-08-17

**Authors:** Marius Alexandru Moga, Oana Gabriela Dimienescu, Cristian Andrei Arvatescu, Aurel Mironescu, Laura Dracea, Liana Ples

**Affiliations:** 1 Department of Medical and Surgical Specialties, Faculty of Medicine, Transilvania University of Brasov, Brasov 500019, Romania; moga.og@gmail.com (M.A.M.); dimienescu.oana@gmail.com (O.G.D.); cristiarv@yahoo.com (C.A.A.); aurel.mironescu@gmail.com (A.M.); 2 Clinical Department of Obstetrics and Gynecology, The Carol Davila University of Medicine and Pharmacy, Bucharest 020021, Romania; liaples@yahoo.com

**Keywords:** natural polyphenols, cervical cancer, carcinogenesis, bioavailability, chemotherapy

## Abstract

Cervical cancer represents the second leading cause of death for women worldwide. The importance of the diet and its impact on specific types of neoplasia has been highlighted, focusing again interest in the analysis of dietary phytochemicals. Polyphenols have shown a wide range of cellular effects: they may prevent carcinogens from reaching the targeted sites, support detoxification of reactive molecules, improve the elimination of transformed cells, increase the immune surveillance and the most important factor is that they can influence tumor suppressors and inhibit cellular proliferation, interfering in this way with the steps of carcinogenesis. From the studies reviewed in this paper, it is clear that certain dietary polyphenols hold great potential in the prevention and therapy of cervical cancer, because they interfere in carcinogenesis (in the initiation, development and progression) by modulating the critical processes of cellular proliferation, differentiation, apoptosis, angiogenesis and metastasis. Specifically, polyphenols inhibit the proliferation of HPV cells, through induction of apoptosis, growth arrest, inhibition of DNA synthesis and modulation of signal transduction pathways. The effects of combinations of polyphenols with chemotherapy and radiotherapy used in the treatment of cervical cancer showed results in the resistance of cervical tumor cells to chemo- and radiotherapy, one of the main problems in the treatment of cervical neoplasia that can lead to failure of the treatment because of the decreased efficiency of the therapy.

## 1. Introduction

Cervical cancer represents the second leading cause of death in women worldwide. In 2012 in Europe there were 58,300 new diagnoses and nearly 24,400 deaths [1]. According to the WHO, Romania holds the first place in Europe regarding cervical cancer mortality, with mortality rates in Romania 2–2.7-fold higher than in other Central and Eastern Europeancountries and 6.3 times higher than the average of the EU countries. A similar situation is also found in underdeveloped regions such as Africa, Asia and South America. This is explained primarily by the fact that in Romania, the diagnosis typcal occurs in the advanced stages of the disease [2]. According to a study by the International Agency of Research for Cancer, it is expected that the mortality due to cancer may double in the next 50 years, rising to 15 million by the year 2020 [3].

Several studies have proven that the cancer risk at the point of specific organs is due to exposure to specific environmental chemicals, biological agents (as Human Papilloma virus, Epstein Barr Virus, HIV1, HCV, *Helicobacter pylori*) or physical agents (such as ionizing radiation, UV) [4,5].

In another report from 2014, it was pointed out that cancer has multifactorial causes, the most important being environmental and life style factors, the etiology being associated with genetic abnormalities and inherited genetic aberrations, both caused by endogenous and exogenous agents [6].

Some epidemiological studies from 2014 have highlighted the importance of the diet and its impact on specific types of neoplasia, raising again interest in the analysis of dietary phytochemicals [7,8]. Phytochemicals are bioactive compounds, including polyphenols, alkaloids, nitrogen compounds or carotenoids, that are classified according to their chemical structure. It had been demonstrated that these compounds can interfere with cell regulation and proliferation, being involved in multiple signaling pathways that are disrupted during tumor initiation, proliferation and propagation. They can be found in vegetables, grains, fruits and other plant products [9,10,11,12].

Daniele del Rio [13] reported in an extensive study from 2013 the importance of polyphenols in the prevention and treatment of different types of cancers, concluding that the anticancer effects of these natural compounds are still not totally known, the studies with promising data indicated that regular consumption of green tea can interfere with the development of cancer. Rodriguez-Mateos et al. [14] also studied the impact of dietary polyphenols on health, highlighting the bioavailability and bioactivity of these compounds. They also observed that clinical trials regarding the effects of polyphenols on cervical cancer are limited, and the most frequent studied compounds are green tea polyphenols and curcumin.

Several studies on human and animal cervical cancer cells proved that polyphenols and their derivatives have antioxidant and anticancer potential. In the last years, the potential chemopreventive and chemotherapy properties of diet-derived agents have raised great interest among esearchers. The polyphenolic compounds have received the most attention for their effects in preventing and treating cervical cancer.

As it can be observed, the global cancer incidence is on an ascending curve, therefore understanding the importance and mechanism of these natural products may improve the health worldwide through the contribution to the development of more and more specific treatment strategies against cancer.

The advantages of polyphenols are, according to Brglez Mojzer el al. [15], their high accessibility, specificity of their response, low toxicity, while the main problems of these compounds are their rapid metabolism and low bioavailability. Recent studies have proposed the nanoformulation of polyphenols in order to prevent their rapid degradation and consequently enable delivery of increased concentrations to the target cells. Another solution to the low bioavailability of these compounds is that combinations of existing anti-cancer drugs with some polyphenols have showed promising results. The purpose of this study was to review the bioavailability, anticarcinogenic properties and the effects of the combination between different polyphenols and anti-cancer drugs used in the treatment of cervical cancer.

## 2. Carcinogenesis—An Overview of the Multifactorial Process

The term of carcinogenesis is defined as a process cauased by an environmental carcinogenic factor (biological, chemical, physical agent) that is characterized by distinct molecular changes that can result ultimately in the formation of a malignant tumor [12,16]. It is a complex multi-step, multi- path and multi-factorial process that requires the participation of innumerable critical molecular players and targeted pathways. In recent years it was pointed out that the process needs an underlying balance of aberrant activation of proto-oncogenes and also an inactivation of tumor suppressor genes [13,14,15,16,17,18].

**Figure 1:**
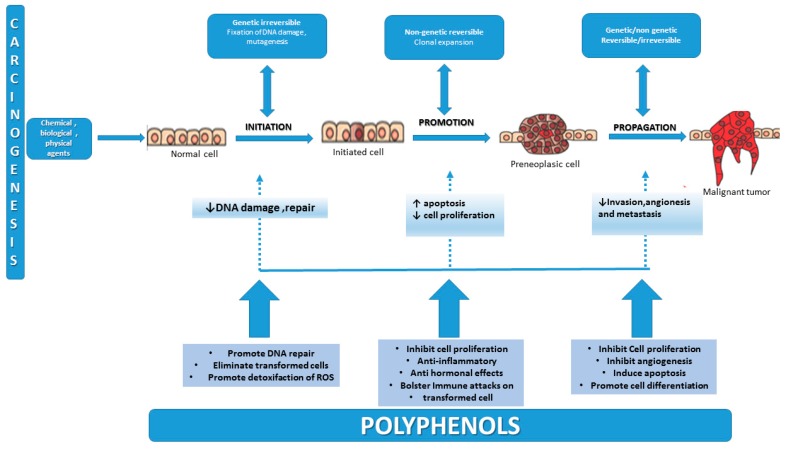
Carcinogenesis—a multifactorial, multi-step process caused by environmental agents—mediated carcinogenesis and steps modulated by chemopreventive polyphenols (adapted after Maru [17] and Kotecha [18]).

The most important changes of the cell during carcinogenesis are initiation, promotion and progression. Figure 1 represents a diagram showing the process of carcinogenesis that can transform a normal cell into a cancerous one and the chemopreventive effects of polyphenols, that can block key events of tumor initiation, promotion and propagation.

The exposure of normal cells to a carcinogenic agent is the first step of carcinogenesis, leading to tumor initiation, an irreversible and rapid process, that results from non-lethal mutations in the cellular DNA. These cells accumulate irreversible genetic modifications with every new cycle of DNA replication. Quail et al. concluded in their study that initiated cells are more immune to inhibitory signals, mediated by cell differentiation inducers and negative growth regulators [19]. The second step, promotion, is a reversible process that consists of selective clonal expansion and proliferation of the initiated cell, having as a result the accumulation of additional mutations, leading to a premalignant cell population, and these cells beginning to undergo the processes of division and propagation [16,17].

Barcellos-Hoff et al. [20] studied the final stage of neoplastic transformation and concluded that progression is an irreversible process, in which the additional genetic mutations lead to new tumor cell clones that possess increased invasive cellular phenotypes and metastatic potential. The research showed that the tumor development is influenced by genetic mutations in two classes of genes: proto-oncogenes (activation of proto-oncogenes results in increased proliferative signals) and tumor suppressor genes (loss or inactivation of these genes), related to the exposure to environmental carcinogens [21]. The accelerated carcinogenesis may be explained by the epigenetic modifications of tumor suppression genes (through DNA methylation) in paraneoplastic tissues [20,22,23].

Even if significant progress was made significant progresses in understanding the molecular mechanism of the malignant cell development, the early detection and treatment of cancer is still a challenging purpose. For this reason, nowadays, preventive interventions had gained the attention of researchers, with important efforts being made in this field. Polyphenols have shown a wide range of cellular effects: they may prevent carcinogens from reaching the targeted sites, support detoxification of reactive molecules, improve the elimination of transformed cells, increase the immune surveillance and the most important factor, they influence the tumor suppressors and inhibit cellular proliferation, interfering in this way with the steps of carcinogenesis [24,25].

## 3. Antioxidants and Their Role in Preventing Cancer

A state in which there is an increased production of reactive oxygen species (ROS) along with a decreased antioxidant cellular stock is specific for oxidative stress. When high levels of ROS are present in the cell, genotoxic damage can occur. Recent studies show a direct correlation between ROSs′ role as secondary messengers in intracellular signaling cascades inducing oncogenesis [26]. Specific to cervical carcinogenesis are high levels of 8-OhdG determining the evolution of lesions from SIL to invasive carcinomas [27]. Changes in lipid peroxidation along with impairment of antioxidant systems (either enzymatic or not) are seen in serum studies of patients with diverse malignant pathologies of the cervix [28,29].

When considering a chemopreventive compound we should mention its specific capabilities of interfering early in the carcinogenesis process in order to eliminate any type of pre-malignant cells that may induce further malignancy [30]. The characteristics of an ideal chemopreventive agent are a selective approach to damaged cells, increased bioavailability in the lesion, multiple mechanisms of action and easy manner of administration. Because of their specificities, dietary compounds are considered the best chemopreventive agents [31,32]. Among these dietary compounds, polyphenols have shown benefit activity as they have anti-proliferative and cytotoxic effects toward tumor cells [32,33]. At 1 g/day their daily intake was shown to be the highest among all classes of dietary anti-oxidants [32,33].

## 4. Natural Sources of Polyphenols, Bioavailability and Effects on Cervical Cancer

Polyphenols are a group of chemical substances from natural and herbal extracts that are arising great interest because of their safe and powerful anticancer effects. Several studies have pointed out that these substances have the property to target viral oncogenes and also to inhibit the deregulation and signaling of gene expression of the host cells [30,34].

These compounds can be divided into two main groups: flavonoids and non-flavonoids [33]. Flavonoids are subdivided into seven subgroups: flavanols, flavanones, flavonols, flavones, isoflavones, anthocyanidins, proanthocyanidins, while the non-flavonoid group includes stilbenes, phenolic acids, lignans and other polyphenols [35,36]. The natural sources, chemical structure and biological activities of dietary polyphenols are summarized in detail in Table 1.

In the following sections are described the most recent research about the anti-cancerogenic activities of the most commonly studied natural polyphenols used for the prevention and treatment of cervical cancer.

### 4.1. Flavonoids

The flavonoids are the most frequent polyphenolic compounds identified in the human diet, being ubiquitously found in plants. They are considered the most important plant pigments involved in flower coloration and being also involved in UV filtration and symbiotic nitrogen fixation. Flavonoids have also other actions as cell cycle inhibitors, physiological regulators and chemical messengers [37]. The chemical structure consists of two aromatic rings (A and B) bound together by three carbon atoms that form an oxygenated heterocycle (ring C). They are subdivided into six subclasses: flavonols, isoflavones, flavones, flavanols (catechins and proanthocyanidins), flavanones and anthocyanidins [37,38].

#### 4.1.1. Flavonols

Are a class of flavonoids widely distributed in numerous fruits and vegetables. Some important food sources are berries, apples, onions, teas, red grapes, broccoli, red wine, curly kale, leeks, broccoli [36,39]. For example, in apples, the flavonols, and particularly quercetin glycosides, are almost exclusively found in the apple peel [40]. The most common flavonols found in the diet are: quercetin, kaempferol, fisetin and myricetin [40]. A study by Somerset et al. identified that the dietary flavonoid sources in the Australian population were 12.53 mg quercetin, 5.6 mg kaempferol and 2.4 mg myricetin [41], the most frequent sources being tea (green and black). In Europe, namely in Spain, in 2010 a study was conducted where the flavonols intake was investigated. Their results showed that 2/3 of ingested flavonols have their origin in onions (mainly quercetin) and the remaining 1/3 of the flavonols intake was from apples, red wine and lettuce [42].

##### Kaempferol

The flavonoid kaempferol (3,5,7-trihydroxy-2-(4-hydroxyphenyl-4*H*-1-benzopyran-4-one) is a low molecular weight yellow compound commonly found in plant-derived foods and in plants used in traditional medicine [49]. The biosynthesis of kaempferol is relatively common in the plant kingdom. In order for kaempferol to form glycosides some sugars (glucose, galactose, rhamnose, and rutinose) are bound to it [50].

Kaempferol is commonly ingested in glycoside form. In many studies, it is reported that highly polar glycosides prevent their absorption, whereas the intermediate polarity of aglycones facilitates it, which makes us believe that for absorbing glycosides hydrolysis to generate aglycones is needed. Aniya et al. [51] reported that glycosides can be absorbed without hydrolysis. Due to the lipophilicity of kaempferol, its absorption is facilitated by passive diffusion, but evidence suggests that it can be absorbed by facilitated diffusion or active transport. In 2010 Lehtonen et al. [52] showed that kaempferol, like other flavonoids is absorbed in the small intestine. Before absorption it can be metabolized under the action of enzymes in the small intestine and further under the action of bacterial flora in the colon, being hydrolyzed into aglycones and sugars. C3 aglycone ring breakage leads to the generation of simple phenolic compounds such as 4-hydroxyphenylacetic acid, phloroglucinol and 4-methylphenol which can be absorbed or excreted in the feces and after absorption are excreted in urine. After absorption, kaempferol is extensively metabolized in the liver to the corresponding glucurono- and sulfo-conjugated forms [52]. A study from 2007 investigated the percentage of kaempferol excreted in urine and found 2.5% of kaempferol while Lehtonen’s study from 2010 found a percentage 0.6% lower [52,53]. In Barve’s et al. study, eight healthy volunteers received endive as a source of kaempferol A, which contained 8.65 mg kaempferol in the form of kaempferol-3-glucuronide (79%), kaempferol-3-glucoside (14%) and kaempferol-3-(6-malonyl)-glucoside (7%).The conclusion of the study was that the mean maximum plasma concentration was 100 nM at 5.8 h after oral ingestion [54]. Also, Radket et al. measured the plasma concentrations of several flavonoids in 48 healthy women and pointed out that the mean intake of kaempferol was 4.7 mg/day and that the corresponding mean plasma concentration was 10.7 nM [55]. In another article by Cao et al. [56] it was reported that an estimated intake of 14.97 mg kaempferol/day led to a plasma concentration of 57.86 nM.

The possible association between the daily intake of food that contains kaempferol and a decreased risk of developing several pathologies as cancer has been evaluated in several epidemiological studies [13,14,50]. For 15 years, numerous scientists, starting with Sanz and his collaborators in 1994 [57] and ending with Verma et al. in 2009 [58] have shown that kaempferol, some glycosides of kaempferol and several kaempferol-containing plants have antioxidant activity not only in vitro but also in vivo. In their papers, we can also see that the most important structural feature of kaempferol, involved in the antioxidant activity is the presence of the double bonds at C3-C4 in conjugation with oxo C4, and the presence of hydroxyl group at C3, C4 and C5 [57,58,59,60,61,62].

##### Quercetin

In the past years, attention was focused on the activity of quercetin, one of the main flavonoids present in fruits and vegetables, in foods derived from plants and beverages. It contains a basic diphenylpropane C6-C3-C6 skeleton. Fruits and vegetables that contain a high quantity of this compound are onions, apples and strawberries [63].

Quercetin is consumed daily by millions of people through nuts, tea, vegetables and herbs in the diet. It is also available as a commercial dietary supplement, and it is now being included in functional foods. Quercetin is generally recognized as safe in oral dosages of 1000 mg/day or in intravenously administered dosages of 756 mg/day. Up to 60% of orally ingested quercetin is absorbed and the average dietary intake of quercetin are somewhere between 6 and 31 mg daily (not including supplement/intravenous use) [64].

The bioavailability of this compound differs among different food sources, being dependent on the type of glycosides containing it. Onions are considered better sources of bioavailable quercetin than apples and tea, which contain rutin and other glycosides [64]. Quercetin is not present as an aglycone, 20%–40% of quercetin being methylated in the 3′-position [65]. Day et al. identified in their study, in which they investigated the exact nature of the metabolites present in plasma after the ingestion of onion, that quercetin-3-*O*-glucuronide, quercetin-3′-*O*-sulfate 3′-*O*-methyl and quercetin-3-*O*-glucuronide are the major conjugates [66].

The degradation of this polyphenol produces mainly 3-methoxy-4-hydroxyphenylacetic (homovanillic acid), 3-hydroxyphenylacetic acid and 3,4-dihydroxyphenylacetic [67]. The reported half-lives range from 11 to 28 h, this characteristic being very important when talking about the elimination of quercetin metabolites because this fact could favor the accumulation in plasma with multiple intakes [68,69].

After absorption, it is metabolized in the small intestine, liver, colon and kidney being conjugated to methyl and sulfate groups and glucuronic acid [70]. The absorption of quercetin also depends on gut microflora. Manach et al. [71] reviewed the bioavailability of this compound and pointed out that the total quercetin derived from the diet is present in plasma at <100 nM, but it can be increased after supplementation (1 month of supplementation with 1 g/day increased plasma concentrations to 1.5 μM) [71,72]. Several articles in the literature have reported a synergistic interaction between quercetin and other bioactive polyphenols [73,74].

#### 4.1.2. Flavones

Flavones are a class of flavonoids that possess a 2-phenylchromen-4-one (2-phenyl-1-benzopyran-4-one) backbone. Among the natural flavones, we can identify apigenin (4′,5,7-trihydroxyflavone), tangeritin (4′,5,6,7,8-pentamethoxyflavone), luteolin (3′,4′,5,7-tetrahydroxyflavone), baicalein (5,6,7-trihydroxyflavone), chrysin (5,7-dihydroxyflavone) and wogonin (5,7-dihydroxy-8-methoxy-flavone). These compounds are mainly found in cereals and herbs, with daily intakes ranging between 20 and 50 mg per day. Among their numerous biological effects, the most important ones are their antioxidant, anti-microbial, estrogenic, anti-proliferative and anti-inflammatory activities [13,14,15,75].

##### Apigenin

Apigenin, with the chemical structure 4′,5,7-trihydroxyflavone, is found in fruits and vegetables such as onions, parsley, oranges, tea, wheat sprouts and chamomile [76,77]. It is considered the most active flavone in plants [13,14,15].

The first extensive study published by Nielsen et al. [78], detected apigenin in urine samples of the participants of a randomized study after having administered a parsley supplement. The urinary excretion rate was estimated to be 0.58% of apigenin [78].

Apigenin compounds are taken up mainly by passive diffusion, because as shown in other studies, only some polyphenol glycosides and aglycones and can be absorbed in the intestine and other glycosides can be hydrolyzed by the microflora in the colon. Other flavonoids found in parsley (quercetin, isorhamnetin and luteolin) interact with apigenin absorption according to the results of Janssen et al. [79]. Other authors [13,14,15,35,36,80] compared the flavonoid absorption, namely between flavones such as apigenin and isoflavones as genistein and observed that intestinal conjugates excreted amounts were 61 nmol/30 min for genistein and 150 nmol/30 min apigenin. Also, apigenin conjugates were excreted much faster than genistein conjugates and apigenin absorption was increased in colon and reduced in the ileum.

Several studies have investigated the anti-carcinogenic effects of this compound, pointing out that apigenin inhibits the growth of cervical cancer cell lines (CaSki, HeLa, and C33A) that cause G1 phase growth arrest through the induction of apoptosis, which was p53 dependent and associated with a marked increase in the expression of p21/WAF1 protein and with the induction of Fas/APO-1 and caspase-3 expression. Apigenin also decreased the expression of Bcl-2 protein, an antiapoptotic factor [81,82].

A study by Czyz et al. [83] pointed out that this polyphenol can interfere at the level of gap junctional coupling and also with the proliferation and survival of the cell. In their study, they exposed HeLa cells and also their connexin43 (Cx43) to apigenin and concluded that this exposure induced an inhibition of translocation of these cells. In the case of low concentrations, the effect of apigenin on the proliferation of the cell was less pronounced, but some modifications were observed in the cell motility (that can mean a reduction of the invasive potential of the HeLa Cx43 cells) [83]. Taken together, these results suggest that apigenin may provide a new therapeutic approach to cervical cancer.

#### 4.1.3. Isoflavones

Isoflavones are most commonly found in vegetables, mostly in soybeans, the major isoflavones being genistein and daidzein [84]. In the literature, it is stated that the levels of isoflavones in soybean vary between 560 and 3810 mg/kg [85]. Soy proteins isolated from soybean contain 466–615 mg isoflavones/kg, while soymilk, bean sprouts and bean curds have up to 2030 mg isoflavone/kg. Izumi et al. reported in their study that the isoflavone aglycones are absorbed in greater amounts and faster than their glycosides in humans [85].

After the ingestion, the isoflavone glucosides are hydrolyzed by bacterial and intestinal mucosal β-glucosidases, releasing the aglycones [84,86], which are then absorbed directly or metabolized by the intestinal microflora in the large intestine into other metabolites [87]. The gut microflora have an important role in the metabolism of isoflavones, in particular in the degradation of daidzein (which is converted to equol—the highly active metabolite and ODMA—the inactive metabolite) [84,86]. The excretion in urine of the isoflavones is as acidic conjugates (glucuronides) and in a small amount as sulphates [88]. After ingestion of food rich in soy compounds, the urinary excretion of genistein and daidzein is typically highest after 7 to 8 h [89].

Yashar et al. [90] studied the hypothesis that genistein can increase the efficacy of radiation used for the treatment of cervix cancer, based on earlier studies that have investigated the effectiveness of genistein as a radiosensitizer against other types of cancer [90,91,92]. Genistein has a heterocyclic diphenolic structure and has been shown to inhibit: (1) tyrosine protein kinases; (2) topoisomerase I and II; (3) protein histidine kinase and (4) 5α-reductase [93,94,95,96]. It also induces G2M cell cycle arrest in some cells. The mechanism that results in the augmentation of cell apoptosis induced by radiation may be due to the disruptive effect of genistein on the cell cycle. The results of the study showed that genistein possesses a dose-dependent inhibition effect on the cervical cell lines and also a radiosensitizing effect on CaSki and Me180 cells. In the case of daidzein, it was reported that it had no effect on radiosensitivity [90].

Populations who typically consume a diet rich in isoflavones have reduced incidence of some types of cancer [97]. Some large epidemiological studies were conducted on Asian populations who consumed approximately 80 mg of total isoflavones per day, compared to European populations who consumed 50 mg/day. The regular dietary intake results in the nanomolar quantities of genistein [98]. In a study from 2006 the effect of combined treatment with genistein was investigated, concluding that the inhibition of the cell growth of cervical cancer cells treated with genistein was time- and dose-dependent. They reported that the treatment with 4 Gy associated with 40 µmol/L of genistein-induced the maximum apoptotic rate, resulting that this combined treatment reduces the possible adverse reactions of the radiotherapy [99]. Guo et al. [100] studied one of the most frequent isoflavones, daidzein and its effect on the treatment of cervical cancer in vitro. Their results showed that at various concentrations, from 6.25 to 100 µmol/L, this compound inhibited the growth of HeLa cells. They concluded that daidzein affected human non hormone-dependent cervical cancer cells in different ways (including cell cycle, cell growth and telomerase activity in vitro). Since treatment of cervical cancer involves multi-modal treatments, new combined approaches need to be developed by clinicians [99,100].

#### 4.1.4. Flavan-3-ols

Flavan-3-ols are represented by catechin, epicatechin, gallocatechin, epigallocatechin, epigallocatechin gallate. A study from 2007 identified [36] catechin and epicatechin as the most commonly found flavan-3-ols originated from fruits (berries, cherries, grapes, plums, apricots), chocolate, red wine and different teas (*Camellia sinensis*) [101,102].

The dietary intake of flavan-3-ols differs among various populations. A study of Somerset et al. [41] pointed out that the daily average intake of catechin and epicatechin in Australia was 9.36 mg and 16.64 mg, respectively, the most common sources consumed being tea (black and green), apples and wine [41]. A similar study performed in Spain also identified wine, tea and apples as the most frequent sources of flavan-3-ols intake [42]. It is well known that tea is frequently consumed in Japan, and the study conducted by Otaki et al. sustained this fact and pointed out that among this population, tea was consumed by 98%, while apples contributed to 1.9% of the daily intake [103]. Even if they are present in many fruits, catechins’ bioavailability has been studied predominantly after ingestion of tea and cocoa.

The only known compound present in plasma in free form in a high proportion is EGCG (77%–90%) [104,105]. The other compounds are conjugated with glucuronic acid ± sulfate groups. The major circulating metabolites of epicatechin are epicatechin-3′-*O*-glucuronide, 4′-*O*-methylepicatechin-5-, 4′-*O*-methylepicatechin-3′-*O*-glucuronide or 7-*O*-glucuronide, 4′-*O*-methylepicatechin and the aglycone epicatechins [106]. Meng et al. [107] identified microbial metabolites in plasma and urine, after intake of tea, accounting for 6%–39% of the ingested epigallocatechin and epicatechins. The actions of catechins could be prolonged by these metabolites, because they appear later in plasma and also they have long half-lives, according to the study of Lee et al. [108]. Catechins are rapidly eliminated, while galloylated catechins are not found in urine [109] because of the preferential excretion of these compounds in bile [105]. Bioavailability differs between different types of catechins. A study that focused mainly on the plasma concentrations of tea catechins showed that galloylation of catechins reduces their absorption and only epigallocatechin was methylated (4′-*O*-methylepigallocatechin was found in an amount of 30%–40% of the total metabolites of epigallocatechin) [105]. In another study, the concentrations of methylated form of epigalocatechin (4′-*O*-methylepigallocatechin) was five times higher than the pure form of epigallocatechin in plasma and three times higher than in urine [110].

##### Epigallocatechin-3-gallate

It is the most bioactive agent found in green tea, extracted from *Camellia sinensis* and it displays characteristics such as antiproliferative, antiangiogenic, antimetastatic, and proapoptotic effects. EGCG shows antiproliferative effects such as the arrest of the cell cycle in the G1 phase preceding chronologically the programmed cell death through apoptosis [111]. Multiple studies have shown a critical role of the estrogen in the HPV-positive mechanism of cervical cancer. Considering that, the overexpression of aromatase results in increased expression and activity of estrogen receptor, inhibition of ERα and aromatase and also inhibition of the growth of the tumor cells [112,113,114].

Moreover, the study conducted by Qiao et al. [115] mentions several other aspects of the use of EGCG: inhibition of HPV E6/E7 expression, ERα and aromatase. Other researchers, Sharma et al. [116] studied HeLa cells treated with EGCG and reported a time-dependent manner of growth inhibition mediated through apoptosis. Along with the EGCG, polyphenol E also derived from green tea had inhibitory effects on cervical cancer. Zou et al. [117] compared in an original study from 2010 the effect of the two substances on the affected cells. Differences were found between two types of cells, respectively squamous cell carcinoma and adenocarcinoma. The inhibition was less when dealing with adenocarcinoma cells concluding thus that the type of cells involved in the development of cervical cancer can modulate the results of the treatment [117].

#### 4.1.5. Flavanones

Flavonoids are potentially involved in the reduction of carcinogenesis by protecting against DNA damage (induction), inhibiting tumor development (promotion) and regressing tumor invasion (proliferation) [13,14,15,35,36]. Anticancer effects of flavonoids have also been established in eukaryotic cell models through selective cytotoxicity, antiproliferative actions and by inducing apoptosis [13,14,15,35,36].

##### Naringenin

Naringenin (NAR) (4,5,7-trihydroxy flavonone) is one of the naturally widespread flavonones present in citrus and grapes [118,119]. Naringenin has various pharmacological effects such as anti-inflammatory, hepatoprotective, antimutagenic, antiatherogenic and anticancer [120,121]. Several studies reported that naringenin induces cytotoxicity and apoptosis in various cancer cell lines [122,123]. But this compound has a poor bioavailability and permeability, instability and extensive first-pass metabolism before reaching the systemic circulation, because of the low aqueous solubility [124]. In order to overcome the obstacles associated with naringegin bioavailability, several researchers developed the nanoparticles of naringenin.

A study conducted in 2011 [125] investigated the anticancer mechanism in cervical cancer cells of naringenin-loaded nanoparticles (NARNP). They reported that only the nanoparticles less than 400 nm in size can cross the vascular endothelium and accumulate at the level of the tumor [126,127]. The results of this study, correlated with other studies indicated that in HeLa cells no cytotoxic or apoptotic effects were found under the influence of naringenin at lower concentrations [128,129]. Only the treatment of HeLa cells with NARNPs produced a dose-dependent cytotoxicity, apoptotic morphological changes, reduced intracellular glutathione levels, alterations in mitochondrial membrane potential, increased intracellular ROS and lipid peroxidation levels and. Therefore, NARNPs have superior advantages over free naringenin in HeLa cells. Ramesh et al. [130] also studied the effects of naringenin on the cervical cancer cell and concluded that this compound inhibits the proliferation through cell cycle arrest at the G2/M phase of human cervical SiHa cells and induce apoptosis through the disruption of DwM.

Other researchers [131] investigated the effect of naringenin on cell growth inhibition and apoptosis through the inhibition of NF-κB-COX-2/caspase-1 pathway HeLa cells. Their study was in accordance with other research that pointed out the antiproliferative effects of naringenin [132,133], reporting that naringenin had important anticancer effects, (by decreasing the cell viability and increasing the number of apoptotic cells and the expression level of cleaved caspase-3 in HeLa cells). It was concluded that treatment of HeLa cells with naringenin attenuated the expression levels of NF-κB p65 subunit, COX-2 and caspase-1 [131,132,133].

#### 4.1.6. Anthocyanidins

Anthocyanins are compounds that are wildly distributed in vegetables and fruits. There are numerous natural anthocyanins, being *O*-glucosylated with various sugar substituents [134]. A review estimated that in the human diet, the daily intake of anthocyanins is approximately 180–250 mg/day [134,135]. The representative types of anthocyanins are cyanidin, malvidin, peonidin, delphinidin and pelargonidin.

In nature, they are found in blueberries, cranberries, and bilberries, black currant, cherry, eggplant, and black rice [136,137]. The biological effects of these compounds are numerous, from reducing the risks of cancers and cardiovascular diseases, anti-inflammatory, chemoprotective and antioxidant properties [138,139]. Another study of anthocyanins showed that they are able to inhibit the low-density lipoprotein oxidation in vitro and decrease serum lipids in cholesterol [140]. Other researchers have discovered that inhibition of cell proliferation of various types of cancer cells and the expressions of cyclooxygenase enzyme can be a consequence of the effects of cyanidin and peonidin [141]. As we discussed before, phenolic compounds have been shown to inhibit tumor cell proliferation, and a study of Fostis et al. supported the notion that anthocyanins have the same effect [142].

Several studies have pointed out the effects of anthocyanins on various cancers. These compounds may inhibit the growth of different cancers by inducing cancer cells toward apoptosis and scavenging ROS. A study from 2006 by Chen et al. [137] investigated the effects of cyanidin 3-glucoside and peonidin 3-glucoside on cell invasion, adhesion, motility and DNA binding activity, MMPs and u-PA expression. They concluded that the inhibition of cancer cell invasion by cyanidin 3-glucoside or peonidin 3-glucoside, may be due to a downregulation of MMP-2, MMP-9 or u-PA expression of various cancer cells [137]. Another study that investigated the mechanism by which cyanidin 3-glucoside acts at the cellular and molecular levels on the proliferation and apoptosis in the cell line HeLa was conducted by Song et al. [143]. They pointed out that the proliferation of HeLa cells was inhibited by black rice anthocyanin and C3G in a dose- and time-dependent manner. The probable mechanism involved in inducing apoptosis was regulation of the expressions of Bax Bcl-2 [143].

### 4.2. Non-Flavonoids

#### 4.2.1. Phenolic Acids

Are an important class of polyphenols being divided into two major groups: hydroxycinnamic acids and hydroxybenzoic acids. They are found in different vegetables like broccoli, spinach, kale, in various fruits as berry fruits and apples, in olive oil or coffee and citrus juices [144].

The most investigated hydroxybenzoic acids are ellagic, gallic and protocatechuic acids while the derivatives of cinnamic acids are mainly represented by caffeic (the most common, which accounts for up to 70% of total hydroxycinnamic acids in fruits), ferulic (cereal grains) and *p*-coumaric acids. These acids esterified with quinic, tartaric acids or carbohydrate derivatives, being rarely found in the free form [13,14,15,35,36].

Because of their low distribution in foods, few bioavailability studies have been carried out on hydroxybenzoic acids The absorption of gallic acid was found under 4-*O*-methylated and *O*-glucuronidated forms [145]. This study investigated the urinary excretion of this compound, concluding that hydroxybenzoic acids were eliminated in an amount of 39.6% of the ingested dose [145].

Another phenolic acid investigated in the literature is ellagic acid, a more complex compound that is found in walnuts, oak-aged wine, strawberry or pomegranate. A study from 2010 has shown that ellagic acid was found without structure modification in human plasma after pomegranate juice consumption [146]. Several studies were conducted regarding the absorption of hydroxycinnamic acid as caffeic acid and ferulic acid. After the ingestion of the free form of hydroxycinnamic acids the absorption was rapid from the stomach or the small intestine being conjugated by the intestinal and/or hepatic detoxification enzymes [147].

The most important acid from the hydroxybenzoic class is gallic acid and the most important source of gallic acid is tea. Shahidi et al. [144] discovered that tea leaves may contain up to 4.5 g/kg fresh tea. Also, gallic acid is widely distributed in various plants and foods, and its various biological effects have been reported in various studies [145].

Several studies have reported that gallic acid may be responsible for the decrease of angiogenesis in vitro and in vivo. Following the intraperitoneal administration of 250 mg *Rubus* extract for 2 days, serum from the rats exhibited significant inhibition of angiogenic initiation and subsequent neovessel growth. The HUVECs were less sensitive to the cytotoxicity induced by gallic acid compared with HeLa and HTB-35 cells at the concentrations of 5, 10 and 15 μg/mL. Based on this data, gallic acid may be considered useful for targeting angiogenesis. Many researchers had studied the action of gallic acid on the EGFR activities and they observed that this compound significantly decreases the phosphorylation of PI3K/AKT and MAPK/ERK signaling pathways, which play key roles in cell proliferation and invasion. Based on this data we can say that gallic acid may be responsible for decreased invasiveness through the suppression of the EGFR/PI3K/AKT and EGFR/MAPK/ERK pathways [145,146].

The hydroxycinnamic acids are more common than the hydroxybenzoic acids and consist mainly of *p*-coumaric, caffeic, ferulic, and sinapic acids. These acids are rarely found in the free form; being found in their bound forms. The bound forms are glycosylated derivatives or esters of quinic acid, shikimic acid, and tartaric acid. Clifford et al. reported in 2000 that caffeic and quinic acid combine to form chlorogenic acid, being found in many types of fruit and in high concentrations in coffee: a single cup may contain 70–350 mg chlorogenic acid [147]. Olthof et al. [148] showed that the esterification of caffeic acid, as in chlorogenic acid, markedly reduced its absorption. In fact, the absorption of chlorogenic acid occurs mainly in the colon, after hydrolysis by microbial esterases. Other studies conducted by Sosulski et al. [149] in 1982 and Van de Putte et al. [150] in 2000 reported that ferulic acid is the most abundant phenolic acid found in cereal grains, which constitute its main dietary source. The ferulic acid content of wheat grain is 0.8–2 g/kg, which may represent up to 90% of total polyphenols [149,150]. Kern et al. [151] measured in 2003 the urinary excretion and plasma concentrations of ferulic acid metabolites after ingestion of breakfast cereals. They deduced from the kinetic data that absorption of ferulic acid from cereals takes place mainly in the small intestine, from the soluble fraction present in cereals. Only a minor amount of ferulic acid linked to arabinoxylans was absorbed after hydrolysis in the large intestine [151].

#### 4.2.2. Curcumin

Curcumin is a polyphenol extracted from *Curcuma longa* Linn, known generally as turmeric. It is a yellow substance from the polyphenol class that had been used as a medical agent and also as a dietary spice. Curcumin (diferuloylmethane) with the chemical structure C_21_H_20_O_6_ is the main curcuminoid found in turmeric, being the most active compound [152].

Curcumin it a hydrophobic polyphenol derived from the rhizome of the herb *Curcuma longa*. It is commonly called diferuloylmethane and it has a wide spectrum of biological and pharmacological activities [153]. The anti-inflammatory and antioxidant activities of curcumin have been observed in vitro studies that showed that the inhibition of lipo-oxygenase and cyclo-oxygenase activities can induce inflammation [154]. Commercial curcumin contains approximately 77% diferuloylmethane, 17% demethoxycurcumin, and 6% bisdemethoxycurcumin. Curcumin has been shown to exhibit antioxidant, anti-inflammatory, antimicrobial, and anticarcinogenic activities. Curcumin is extremely safe, even at very high doses. For example, in some animal models or human studies it was proved that curcumin, when taken as high as 12 g per day, is well tolerated. The pharmacological safety and efficacy of curcumin make it a potential compound for treatment and prevention of a wide variety of human diseases, including cancer [153,154].

To increase the bioavailability, better permeability and longer circulation it was necessary to add different components such as nanoparticles, liposomes, micelles and phospholipid complexes [155] After that, various pharmacological studies revealed that curcumin is safe and effective in treatment and prevention of a wide variety of human diseases including cancer*.* Wahlstrom et al. [156] were the first one to report that after oral administration of 1 g/kg of curcumin in rats, in blood plasma of rats were found negligible amounts of curcumin, being a consequence of the poor absorption from the gut. Later, in another study, when curcumin was given orally at a dose of 2 g/kg to rats, a maximum serum concentration of 1.35 ± 0.23 µg/mL was observed at 0.83 h, whereas in humans the same dose of curcumin resulted in either undetectable or extremely low (0.006 ± 0.005 µg/mL at 1 h) serum levels [157].

Ryu et al. [158] also studied the bioavailability of curcumin in different mice organs. Intravenous injection of curcumin in mice found persistently accumulation in the liver and spleen while lung uptake were found to decrease with time. A study using radioactive curcumin and piperine in mice, observed that initial brain uptake of curcumin increased by 48% relative to that without piperine, although other organ uptakes were almost similar to those without piperine [159].

The effect of curcumin in HPV-associated cells was found to involve the downregulation of viral oncogenes, NF-jB and AP-1 [160,161]. Similarly, a major metabolite of curcumin called THC increased the sensitivity of vinblastine, mitoxantrone, and etoposide in a drug-resistant human cervical carcinoma cell line. In a phase I clinical trial, a daily 0.5–12 g dose of curcumin taken orally for 3 months resulted in the histologic improvement of precancerous lesions in one out of four patients with uterine cervical intraepithelial neoplasms. Some in vitro studies over the past decade have shown that curcumin and a curcumin-paclitaxel conjugate had therapeutic effects in ovarian cancer cell lines. In the in vivo study, tumors were grown by orthotopic injection of cells and 1 week after orthotopic implantation animals were treated with curcumin (500 mg/kg/day, gavage) alone or in combination with docetaxel (35–50 μg/animal/week, i.p.) for 4 weeks [159,160,161,162]. Curcumin alone resulted in 49%–55% reductions in mean tumor growth compared with controls whereas when combined with docetaxel 77% reductions in mean tumor growth compared with controls was obtained for curcumin in normal ovarian tumor models [163,164].

Strimpakos et al. [165] reported in their study that curcumin inhibits carcinogenesis through various mechanisms including antioxidant, anti-inflammatory, proapoptotic, anti-angiogenic and immunomodulatory properties. The result of this mechanism is the pleiotropic effect on cell-signaling pathways and genes, at different levels [166,167,168]. A study in vitro of human cervical cancer cells performed by Aggarwal in 2009 concluded that curcumin has the property to downregulate COX-2 [169].

Its effect on NF-κB activity in cervical cancer cells was observed in 2005 when increased sensitization to cisplatin treatment for the SiHa cell line firstly treated with curcumin was noticed [170]. Moreover, according to several studies, curcumin is involved in the suppression of all three stages of carcinogenesis: initiation, promotion and progression, therefore it is the perfect candidate for sensitizing difficult to treat cells. Curcumin shows sensitizing effects for cervical cells treated subsequently with taxol, acting on down-regulation of NF-κB and serine kinase AKT pathway [171,172]. Similarly, curcumin administered during paclitaxel treatment acts as a downregulator of paclitaxel on preo-survival pathways linked to NF-κB and Akt activation [173].

The chemotherapeutic activity of curcumin was investigated in a phase I clinical study performed by Cheng et al. in a group of patients with preinvasive malignant or high-risk premalignant affections [174]. A dose of 1 to 8 g of curcumin was administered daily for 3 months, with the histological improvement of the premalignant lesions in 25% of patients with cervical intraepithelial lesions being noted [174].

Finally, another important aspect of curcumin’s activity is the radioprotective effect on normal cells and radiosensitizing effects on cancer cells, even if the mechanisms of these opposing actions are not fully known. Some authors have suggested that the ability of curcumin to reduce oxidative stress and inhibit the transcription of genes related to oxidative stress and inflammatory responses may afford protection against the harmful effects of radiation, while the radiosensitizing activity might be due to the upregulation of genes responsible for cell death [174,175,176,177,178].

A study of Maher et al. from 2011 showed that curcumin has a molecular target on down-regulating HPV-18 transcription, inhibiting AP-1 binding activity and reversing the expression of *c*-fos and fra-1 in HeLa cells [177]. More important, curcumin was shown to decrease the transformation of the phenotype and to stop the cellular growth of malignant cells [178].

Taking into account the information presented regarding the role of curcumin on malign modified cervical cells is clear that curcumin shows important potential in treating and preventing cervical cancer, but further studies need to be done to confirm this role.

#### 4.2.3. Stilbenes

Stilbenes are identified in the human diet in low quantities, resveratrol being the most representative and extensively studied over the past years. Chemically known as 5-((*E*)-2-(4-hydroxyphenyl)ethenyl)benzene-1,3-diol; C_14_H_12_O_3_), resveratrol is a polyphenolic flavonoid found in the seeds and skins of grapes, red wine, mulberries, peanuts, and rhubarb. Its beneficial effects are seen in those who consume mostly fresh fruits and vegetables as it has been demonstrated that this kind of diet sustains a long healthy life through various processes such as activation of intracellular pathways [13,14,15,35,36,179].

Resveratrol is shown to act as an anti-tumoral factor interacting with the Fa pathway, Rb-E2F/DP pathway, NF-κB and AP-1 transcriptional factors [179]. In some epidemiological studies, the absorption of resveratrol using a moderate intake of wine (25 mg) was investigated, but the authors didn’t identify unmetabolized resveratrol in plasma, concluding that resveratrol has a very low oral bioavailability [180,181]. The absorption of this compound was unusually high for a dietary polyphenol, mostly because of the poor aqueous solubility [13,14,15,35,36].

Lu et al. reviewed in 2007 the accumulation of resveratrol in tissue, being known that this compound accumulates mostly in tissues as compared with plasma [181]. A high amount of resveratrol accumulation has been found in intestinal tissue in mice [182]. As reviewed by Hsu et al. in 2009 [183] resveratrol had demonstrated to inhibit proliferation and induce autophagy and apoptotic death in cervical cancer cells. In cervical cancer cells, after a treatment with resveratrol, it was pointed out that autophagy responses increased significantly after 6 h. Other several studies have been conducted in order to investigate the effects of resveratrol on the cervical cancer cells. A study from 2012 [184] pointed out that this polyphenolic compound determines a reduction in the generation of ROS and induces invasion and migration in HeLa cells. It also decreases the expression and the enzymatic activity of MMP-9. The conclusion of the study was that resveratrol inhibits NF-κB and AP-1 transactivation suppressing the transcription of MMP-9, leading to suppression of migration and invasion of cervical cancer cells.

Studies conducted by Zoberi and Garcia Zepeda et al. [185,186] and Kramer et al. [187] confirm the role of resveratrol in inducing an early *S*-phase arrest, being involved thus in the regulation of the cell cycle progression. Many other researchers brought proof of the role of resveratrol on COX. Among these scientists, Garcia Zepeda et al. [186] noticed a suppression of COX-2 when using resveratrol on cervical cancer cells. Another anti-tumoral mechanism that is present in resveratrol is the inhibition of metalloproteinases (MMP). A study conducted on CaSki cervical cancer cells showed a decreased expression of MMP-9 after exposure to resveratrol. The inhibition of AKT and ERK1/2 in cervical cancer cells thus decreasing the angiogenic activity, destabilization of lysosomes, induction of autophagy increased cytosol translocation are other mechanisms of resveratrol [186].

## 5. Studies of Polyphenols and Cervical Cancer

Polyphenols showed marked biological abilities both in vitro and in vivo experiments: scavenge free radicals, induce apoptosis, inhibit cell proliferation and angiogenesis and exhibit phytoestrogen activity. Moreover, they suppress the NF-κB and the activating protein (AP-1), inhibit the mitogen-activated proteins ( MAPKs), the protein kinase and growth factor receptor-mediated pathways, are involved in cell cycle arrest and possess anti-inflammatory properties [13,14,15,35,36].

Despite the promising in vitro study results, clinical trials focused on the anticarcinogenic effects of polyphenols are missing. To date, most of the available clinical studies on humans focused on polyphenols’ bioavailability, pharmacokinetics and metabolism and only a few reports showed that polyphenols could represent a promising agent in the treatment of cancerogenesis. Several clinical studies regarding the anticarcinogenic effects of polyphenols on cervical cancer were conducted. EGCG and curcumin were the most investigated compounds.

Table 2 summarize the studies found in the literature regarding the anticarcinogenic properties of different classes of polyphenols, the attention being focused on their activity and mechanism of action.

In a phase I study [174] the effects of curcumin in patients with cervical cancer were investigated. The results pointed out that in 25% of the patients under treatment, curcumin showed a chemopreventive effect. They also investigated the bioavailability and toxicology of this compound, concluding that the safe dose of curcumin is up to 8 mg by day. Talwar et al. [188] tested curcumin on 280 women with HPV infections, in order to investigate the safety and efficacy against HPV infection.

A phase II randomized study from 2013 [189] also evaluated the efficacy of curcumin in cervical cancer. Vaginal capsules and Basant polyherbal vaginal cream (containing extracts of curcumin, reetha, amla and aloe vera) were used on 287 women. Their results showed that HPV clearance rate in case of using Basant vaginal cream was 87.7% while the use of curcumin vaginal capsule showed a rate of clearance of 81.3%.

The second most investigated polyphenolic compound in the treatment of cervical cancer lesions was EGCG. The researchers [190] investigated the clinical efficacy of EGCG and also other green tea compounds (poly E capsule 200 mg EGCG, 37 mg epigallocatechin, and 31 mg epicatechin) in patients with HPV cervical lesions. Their results pointed out that 60% of patients under EGCG capsule therapy, 50% under poly E capsule therapy, 74% under poly E ointment therapy and 75% under poly E ointment plus poly E capsule therapy showed a response, mainly a 69% response rate as compared with a 10% response rate in untreated controls. They concluded that green tea compounds used orally ± vaginally are effective in the treatment of HPV-related cervical lesions.

## 6. Combinations of Polyphenols and Cervical Anti-Cancer Therapy

It is important to also point out the effects of polyphenols combined with different therapies used in the treatment of cervical cancer. The resistance of cervical tumor cells to chemo- and radiotherapy is one of the main problems in the treatment of cervical neoplasia, leading to failure of the treatment because of the decreased efficacy of the therapy [203]. In the following section, we will point out the effects of the combinations of polyphenols with chemotherapy and radiotherapy used in the treatment of cervical cancer. Several recent studies have proven that the combination of polyphenols with cervical cancer therapies may lead to sensitization of cervical cell cancer to chemo- and radiotherapy and also it can minimize the toxicity of these therapies [203,204].

### 6.1. Polyphenols and Chemotherapy

One of the most used chemotherapeutic agents for the treatment of cervical cancer is cisplatin, but chemoresistance to this drug is a major problem worldwide. It has remained a major limitation of cisplatin-based chemotherapy [203,204]. A possible strategy to overcome this resistance is the combination of this drug with natural flavonoids.

A study from 2013 conducted by Singh et al. [203] reported that green tea compounds such as EGCG are capable of chemosensitizing cervical cancer cells (HeLa, SiHa) to cisplatin through enhancement in cytotoxicity and also by induction of apoptosis due to excessive ROS generation. Another flavonoid compound that was reported to sensitize cisplatin is wogonin, being proved that the main effect is the synergistic cytotoxicity with enhancement of apoptotic death in human cervical cancer cells [205]. Another adjuvant flavonoid of cisplatin, studied in the literature in the last years was quercetin. The study of Jakubowicz-Gil et al. [206] concluded that this compound helps HeLa cells to become more sensitized to apoptosis caused by cisplatin.

Another drug used in chemotherapy of cervical cancer is paclitaxel but it is well known that this agent has harmful side effects. Therefore possible combinations of paclitaxel with flavonoids, that can decrease the side effects were investigated. Xu et al. [207] reviewed apigenin and its effects on human cervical cancer cells and concluded that this compound can sensitize HeLa cells to paclitaxel-induced apoptosis thought to enhance the intracellular ROS accumulation.

The third agent used in chemotherapy for cervical cancer is epirubicin, but the main problem is also the resistance. Lo et al. [208,209] extensively researched the effects of isoflavones on chemo- sensitizing HeLa cells. He identified 7,3′,4′-trihydroxyisoflavone (7,3′,4′-THIF) and formononetin, as being able to increase the cytotoxicity of epirubicin, concluding that these compounds can be used as adjuvants to increase the chemosensitivity of epirubicin in HeLa cells.

### 6.2. Polyphenols and Irradiation

One of the most important treatments for cervical cancer widely used is radiation, but as in the case of chemotherapy, radioresistance is also considered a major issue. Quercetin is also used for radiosensitizing the human cervical cancer cells. A study of Lin et al. showed a radiosensitizing enhancement ratio of 1.65 in the case of a combination of radiotherapy and quercetin [210].

Other studies reported genistein as a potent radiosensitizing agent. It can enhance the radiosensitivity of HeLa cells through modulating cell cycle progression and increasing apoptosis. Shin et al. [211] reported that genistein behaves as a radiosensitizer also in another human cervical cancer cell line as CaSki leading to inducing apoptosis via ROS modulation and a decrease in cellular viability due to the downregulation of E6 and E7 expression.

## 7. Conclusions and Future Perspectives

Cervical cancer is 2nd cancer among women worldwide, especially in countries in development, as it is the case of Romania. Even if the screening methods are available for the purpose of early diagnosis and treatment of this neoplasia, it still represents a major problem of public health.

Various natural polyphenols have shown cytotoxic effects on human cervical cancer cell lines, providing new perspectives in drug development again cervical cancer. Natural polyphenols are bioactive compounds that are demonstrated to have anticarcinogenic properties, in addition to the anti-inflammatory and antioxidant ones. In the last years, the potential chemopreventive and chemotherapy properties of diet-derived agents raised great interest for the researchers.

From the studies reviewed in this paper, we concluded that dietary polyphenols hold potential in the prevention and therapy of cervical cancer because they interfere in carcinogenesis (in the initiation, development and progression) by modulating the critical processes of cellular proliferation, differentiation, apoptosis, angiogenesis and metastasis. Specifically, polyphenols inhibit the proliferation of HPV cells, through induction of apoptosis, growth arrest, inhibition of DNA synthesis and modulation of signal transduction pathways. The anticarcinogenic activity of natural polyphenols is different depending on the origin of cervical carcinoma or cellular type line. The current knowledge about the cytotoxic mechanism of polyphenols is still not fully known, only a few clinical studies have been conducted focusing on the anticarcinogenic effects of dietary polyphenols.

Another important problem of the treatment of cervical cancer is the resistance to chemotherapy and radiotherapy. In this review we observed that several polyphenols are able also to sensitize cervical cancer cells to conventional chemo- and radiation therapy. This combined approach could improve the efficiency of standard therapies and allow to decrease the doses of chemotherapy drugs and irradiation leading to reduce the adverse side effects. A major challenge of cancer prevention is to integrate the new molecular findings into clinical practice. The modification of these molecules using nanotechnology could be a solution to improve their low bioavailability and also aid in targeting them to specific tissues. Studies that focus on the natural polyphenols should continue to provide researchers an improved understanding of polyphenols absorption, distribution, role in the anti-cancer mechanism. From this review, we concluded that natural polyphenols could become key role players in future treatment and prevention of cervical cancer. We summarized in this article some of the polyphenolic compounds that have been studied until now for their possible anti-cancer therapeutic properties. It is crucial to continue these studies for searching therapeutic drugs from natural resource as well as investigating their mechanism of action in cervical tumor cells.

## Figures and Tables

**Table 1:** Sources of polyphenols, chemical structure and their biological activities [13,14,15,35,36,43,44,45,46,47,48].

Compound	Chemical Structure	Dietary Sources	Biological Effects
**Flavonols**			
Epicatechin	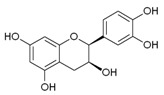	Apple, berries, grapes, red wine, green and black tea, chocolate	Antioxidative, anti-proliferative, pro-apoptotic, antiangiogenic, suppression of growth and invasion, anti-inflammatory, antimetastatic, antimutagenic, inhibition of telomerase activity and lipid peroxidation, modulation of estrogen activity, modulation and reversal of epigenetic changes
Catechin	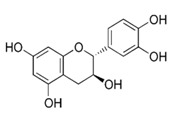	Red wine, broad beans, black grapes, apricots, tea, strawberries	Antioxidative, anti-proliferative, pro-apoptotic, antiangiogenic, inhibition of tumor growth, anti-inflammatory, suppression of growth and invasion, pro-oxidative
**Flavones**			
Apigenin	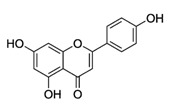	Parsley, celery, celeriac, and chamomile tea	Antioxidative, anti-mutagenic, anti-inflammatory, anti-viral, inhibition of tumor growth, pro-apoptotic, suppression of tumor progression, anti-invasive, antiangiogenic, antimetastatic, anti-proliferative, modulation of epigenetic changes
Luteolin	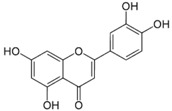	Celery, broccoli, green pepper, parsley, thyme, dandelion, chamomile tea, carrots, olive oil, peppermint, rosemary, navel oranges, and oregano	Anti-inflammatory, anti-mutagenic, anti-carcinogenic
Chrysin	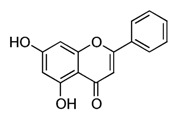	Passion flowers, chamomile, honeycomb	Anti-proliferative, anti-anxiety, anticonvulsant, antioxidant, anti-inflammatory
**Flavonols**			
Quercetin	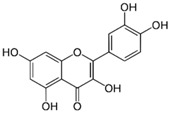	Onions, broccoli, apples, apricots, berries, nuts, seeds, tea, wine, cocoa	Antioxidative; pro-oxidative, antiviral, inhibition of tumor formation and migration, pro-apoptotic, anti-proliferative, antimetastatic, anti-angiogenic, inhibition of lipid peroxidation, reduction of tumor incidence and multiplicity, prevention of GJIC inhibition, modulation of epigenetic changes
Kaempferol	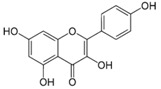	Apples, grapes, tomatoes, green tea, potatoes, onions, broccoli, Brussels sprouts, squash, cucumbers, lettuce, green beans, peaches, blackberries, raspberries, and spinach	Antioxidant, anti-viral, antibacterial, antiproliferative, anti-inflammatory
**Flavanones**			
Naringenin	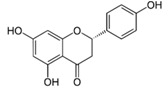	Grapefruit, oranges, and tomatoes	Anti-oxidative, anti-inflammatory, anti-metastatic, delayed tumor development, reduction of tumor incidence, anticarcinogenic, lipid-lowering, superoxide scavenging, anti-apoptotic, metal chelating
**Anthocyanins**			
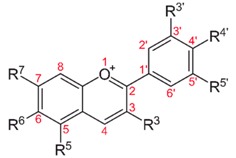		Blueberry, cranberry, bilberry; black raspberry, red raspberry, blackberry; blackcurrant, cherry, eggplant (aubergine) peel, black rice, Concord grape, red cabbage, and violet petals. Red-fleshed peaches and apples contain anthocyanins	Anti-inflammatory, anti-edema, antiproliferative, antioxidant, antiangiogenic, antimetastatic
**Proanthocyanidins**			
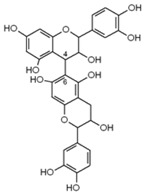		Cinnamon, aronia fruit, cocoa beans, grape seed, grape skin, red wines, bilberry, cranberry, black currant, green tea, black tea	Antioxidant, antiproliferative, antibacterial, anti-inflammatory
**Isoflavones**			
Daidzein	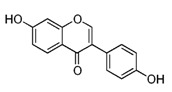	Kwao Krua, Kudzu, Maackia amurensis cell cultures, tofu	Antioxidant, estrogenic and anti-estrogenic effects
Genistein	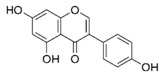	Lupin, fava beans, soybeans, kudzu, psoralea, coffee	Antioxidative, anti-invasive, anti-inflammatory, anti-metastatic, delay/repression of tumor development/growth, reduction of tumor multiplicity and volume, pro-apoptotic, antiproliferative, estrogenic activity, prevention of GJIC inhibition, modulation of epigenetic changes
**Stilbenes**			
Resveratrol	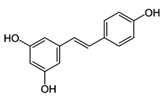	Skin of grapes, blueberries, raspberries, mulberries	Antioxidative, anti-inflammatory, anti-cyclooxygenase, antiproliferative, proapoptotic, antiestrogenic, modulation of lipid metabolism, inhibition of platelet aggregation
**Lignans**			
Secoisolari-ciresinol	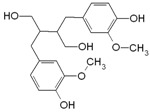	Flax, sunflower, sesame, pumpkin seeds	Antioxidant, anti-inflammatory, antiproliferative, anticarcinogenic
**Phenolic Acids**			
Benzoic acids (Gallic acid)	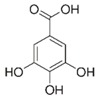	Gallnuts, sumac, witch hazel, tea leaves, oak bark	Antioxidative, pro-oxidative, anti-inflammatory, antibacterial, antiviral, anti-melanogenic, antimutagenic, suppression of tumor growth, anti-invasive, antiproliferative, inhibition of tumorigenesis, anti-angiogenic, modulation of androgen receptor
Cinnamic acids	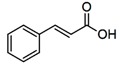	Oil of cinnamon, balsams such as storax, shea butter	Antioxidative, antimicrobial, anti-inflammatory, antiproliferative
**Tannins**			
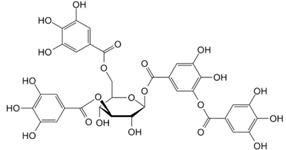		Grape skins, seeds and stems, cranberries, strawberries, blueberries, hazelnuts, walnuts, pecans, Cloves, tarragon, cumin, thyme, vanilla, and cinnamon	Antimicrobial activities, Antitumor activities, Inhibition of the mutagenicity of carcinogens, Inhibition of tumor promotion
**Other Polyphenols**			
Curcumin	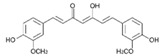	Turmeric	Antioxidative, anti-angiogenic, anti-adhesive, tumor growth suppressive, antiproliferative, proapoptotic, antimetastatic, anti-inflammatory, modulation and reversal of epigenetic changes
Rosmarinic acid	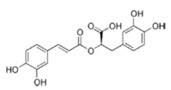	Basil, lemon balm, rosemary, marjoram, sage, thyme, peppermint	Antioxidative, reduction of HCA formation, modulation of epigenetic changes
6-Gingerol	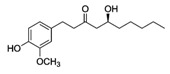	Fresh ginger	Antioxidative, anti-inflammatory

**Table 2:** Comparative anticarcinogenic properties of polyphenols in cervical cancer [12,13,14,15,35,36,191].

Class	Chemical Constituent	Study Type	Cell Type	Activity	Mechanism of Action	References
Flavanols	Quercetin	In vitro	HeLa	Antiproliferation Induction of apoptosis	Induction of G2/M phase cell cycle arrest and mitochondrial apoptosis; inhibition of anti-apoptotic AKT and Bcl-2 expression	[192]
	Kaempferol	In vitro	HeLa	Antiproliferation	Induction of G2/M phase growth arrest, decrease of cyclin B1 and CDK1, inhibition of NF-κB nuclear translocation, upregulation of Bax and downregulation of Bcl-2	[193]
	Fisetin	In vitro/in vivo	HeLa	Antiproliferation Induction of apoptosis	Significantly reduced tumor growth; Activation of the phosphorylation ERK1/2, inhibition of ERK1/2 by PD98059, activation of caspase-8/-3 pathway	[194]
Flavones	Apigenin	In vitro	HeLa	cell cycle arrest and apoptosis	Decreased in the protein expression of Bcl-2 protein; induced p53 expression; down regulation of Bcl-2 expression	[82]
	Luteolin	In vivo	HeLa	Induction of apoptosis and tumor growth	Luteolin sensitized HeLa cells to TRAIL-induced apoptosis by both extrinsic and intrinsic apoptotic pathways	[195]
Isoflavones	Daidzein	In vitro	HeLa	Inhibition of tumor growth	Expression of human telomerase catalytic subunit mRNA decreased. Affected cell growth, cell cycle and telomerase activity in vitro	[100]
	Genistein	In vitro	CaSki	inhibits growth of cervical cancer cells	Inhibition of Mcl-1 correlated with increase in radiosensitivity in Me180 cells. Activated pAKT (Thr 308) was inhibited enhancement of the radiation effect that may be partially mediated by G(2)M arrest, Mcl-1 and activation of the AKT gene. migration-inhibition in a time-dependent manner by modulating the expression of MMP-9 and TIMP-1	[91,92,93]
Flavanones	Naringenin	In vitro	SiHa	Antiproliferation Induction of apoptosis	Induction of apoptosis through both death-receptor and mitochondrial pathways	[130,133]
	Hesperetin	In vitro	SiHa	reduction in cell viability and the induction of apoptosis	Attenuation of mitochondrial membrane potential with increased expression of caspase-3, caspase-8, caspase-9, p53, Bax, and Fas death receptor and its adaptor protein Fas-associated death domain-containing protein (FADD) induced apoptosis was confirmed by TUNEL and Annexin V-Cy3	[196,197]
Anthocyanidins	Cyanidin	In vitro	HeLa	Antiproliferative	Induced the accumulation of peroxides. inhibited HeLa human cervical tumor cell proliferation and increased generation of reactive oxygen species	[143]
Flavan-3-ols	EGCG	In vitro	HeLa	Antiproliferation	Combination of EGCG with RA induced apoptosis and inhibited telomerase activity	[198]
Phenolic acids	Gallic acid	In vitro	HeLa	Induction of apoptosis	Induction of cell death via apoptosis and/or necrosis was accompanied by ROS increase and GSH depletion	[146]
Stilbens	Resveratrol	In vitro	SiHa, HeLa, C-33A	Antiproliferation	Suppression of C-33A, SiHa and HeLa cells growth through induction of cell apoptosis	[199]
Tannins	Emodin	In vitro	Bu 25TK	Antiproliferative Induction of apoptosis	Inhibited DNA synthesis and induced apoptosis by increased nuclear condensation, annexin binding and DNA fragmentation apoptotic pathway is caspase-dependent	[200]
Curcuminoids	Curcumin	In vitro	HeLa SiHa CaSki	Antiproliferation Induction of apoptosis	Upregulation of Bax, AIF, release of cytochrome c and downregulation of Bcl-2, Bcl-XL, COX-2, iNOS and cyclin D1	[201]
Lignans	methylenedioxy lignan	In vitro	HeLa	Antiproliferative Induction of apoptosis	Inhibiting telomerase and activation of c-*myc*and caspases leading to apoptosis induces apoptosis by bcl-2 suppression and activation of caspases	[202]

## Abbreviations

The following abbreviations are used in the manuscript:

8-OhdG	8-Oxo-2′-deoxyguanosine
ACE-c	Aqueous cinnamon extract
AKT	Protein Kinase B
AP-1	Activating protein
Bak	Bcl-2 homologous antagonist/killer
Bax	Bcl-2-like protein 4
Bcl-2	B-cell lymphoma 2
CaSki	Human cervical carcinoma cell line
Cdk1	Cyclin-dependent kinase 1
CIN	Cervical intraepithelial neoplasia
COX-2	Cyclooxigenaze 2
Cx43	Connexin43
DR5	Death receptor 5
EGCG	Epigallocatechin gallate
ERK1/2	Extracellular signal-regulated kinases
ERα	Estrogen receptor alpha
EU	European Union
Fas/APO-1	Fas receptor/Apoptosis antigen 1
Her-2	Human Epidermal Growth Factor Receptor 2
HIV1	Human Immunodeficiency Virus
HPV	Human Papiloma Virus
HSIL	High squamous intraepithelial lesions
JNK	c-Jun *N*-terminal kinase
LSIL	Low Squamous Intraepithelial Lesions
MAPK	Mitogen-Activated Protein Kinase
MAPKs	Mitogen-Activated Proteins
MMP	Metalloproteinases
NF-κB	Nuclear Factor-Kappab
p65	Transcription factor p65
ROS	Reactive oxygen species
SIL	Squamous Intraepithelial Lesions
TRAIL	Tumor Necrosis Factor-Related Apoptosis-Inducing Ligand)
UV	Ultraviolet
WHO	World Health Organization

## References

[1] Ferlay J., Steliarova-Foucher E., Lortet-Tieulent J., Rosso S., Coebergh J.W.W., Comber H., Forman D., Bray F. (2013). Cancer incidence and mortality patterns in Europe: Estimates for 40 countries in 2012. Eur. J. Cancer.

[2] Arbyn M., Antoine J., Mägi M., Smailyte G., Stengrevics A., Suteu O., Valerianova Z., Bray F., Weiderpass E. (2011). Trends in cervical cancer incidence and mortality in the Baltic countries, Bulgaria and Romania. Int. J. Cancer.

[3] International Agency for Researvh on Cancer Globocan 2012: Estimated Cancer Incidence, Mortality and Prevalence Worldwide in 2012. http://www.globocan.iarc.fr.

[4] Tomatis L., Huff J., Hertz-Picciotto I., Sadler D.P., Bucher J., Boffetta P., Axelson O., Blair A., Taylor J., Stayner L. (1997). Avoided and avoidable risks of cancer. Carcinogenesis.

[5] Cogliano V.J., Baan R., Straif K., Grosse Y., Lauby-Secretan B., El Ghissassi F., Bouvard V., Benbrahim-Tallaa L., Guha N. (2011). Preventable exposures associated with human cancers. J. Natl. Cancer Inst..

[6] Aleksandrova K., Pischon T., Jenab M., Bueno-de-Mesquita H., Fedirko V., Norat T., Romaguera D., Knuppel S., Boutron-Ruault M.-C., Dossus L. (2014). Combined impact of healthy lifestyle factors on colorectal cancer: A large European cohort study. BMC Med..

[7] Garcia D.O., Thomson C.A. (2014). Physical activity and cancer survivorship. Nutr. Clin. Pract..

[8] Howes M.J., Simmonds M.S. (2014). The role of phytochemicals as micronutrients in health and disease. Curr. Opin. Clin. Nutr. Metab. Care.

[9] Priyadarsini R.V., Nagini S. (2012). Cancer chemoprevention by dietary phytochemicals: Promises and pitfalls. Curr. Pharm. Biotechnol..

[10] Surh Y.J. (2003). Cancer chemoprevention with dietary phytochemicals. Nat. Rev. Cancer.

[11] Lee K.W., Bode A.M., Dong Z. (2011). Molecular targets of phytochemicals for cancer prevention. Nat. Rev. Cancer.

[12] Loeb L.A., Harris C.C. (2008). Advances in chemical carcinogenesis: A historical review and prospective. Cancer Res..

[13] Del Rio D., Rodriguez-Mateos A., Spencer J.P., Tognolini M., Broges G., Crozier A. (2013). Dietary (poly)phenolics in human health: Structures, bioavailability, and evidence of protective effects against chronic diseases. Antioxid. Redox Signal..

[14] Rodriguez-Mateos A., Vauzour D., Krueger C.G., Shanmuganayagam D., Reed J., Calani L., Mena P., Del Rio D., Crozier A. (2014). Bioavailability, bioactivity and impact on health of dietary flavonoids and related compounds: An update. Arch. Toxicol..

[15] Brglez Mojzer E.B., Knez Hrnčič M., Brglez Mojzer E., Knez Hrnčič M., Škerget M., Knez Ž., Bren U. (2016). Polyphenols: Extraction methods, antioxidative action, bioavailability and anticarcinogenic effects. Molecules.

[16] Tokarz P., Blasiak J. (2014). Role of mitochondria in carcinogenesis. Acta Biochim. Pol..

[17] Maru G.B., Hudlikar R.R., Kumar G., Gandhi K., Mahimkar M.B. (2016). Understanding the molecular mechanisms of cancer prevention by dietary phytochemicals: From experimental models to clinical trials. World J. Biol. Chem..

[18] Kotecha R., Takami A., Espinoza J.L. (2016). Dietary phytochemicals and cancer chemoprevention: A review of the clinical evidence. Oncotarget.

[19] Quail D.F., Joyce J.A. (2013). Microenvironmental regulation of tumor progression and metastasis. Nat. Med..

[20] Barcellos-Hoff M.H., Lyden D., Wang T.C. (2013). The evolution of the cancer niche during multistage carcinogenesis. Nat. Rev. Cancer.

[21] Letelier P., Brebi P., Tapia O., Roa J.C. (2012). DNA promoter methylation as a diagnostic and therapeutic biomarker in gallbladder cancer. Clin. Epigenet..

[22] Aoi J., Endo M., Kadomatsu T., Miyata K., Ogata A., Horiguchi H., Odagiri H., Masuda T., Fukushima S., Jinnin M. (2014). Angiopoietin-like protein 2 accelerates carcinogenesis by activating chronic inflammation and oxidative stress. Mol. Cancer Res..

[23] Solomon H., Brosh R., Buganim Y., Rotter V. (2010). Inactivation of the p53 tumor suppressor gene and activation of the Ras oncogene: Cooperative events in tumorigenesis. Discov. Med..

[24] Collins A.R., Azqueta A., Langie S.A. (2012). Effects of micronutrients on DNA repair. Eur. J. Nutr..

[25] Royston K.J., Tollefsbol T.O. (2015). The epigenetic impact of cruciferous vegetables on cancer prevention. Curr. Pharmacol. Rep..

[26] Liou G.Y., Stors P. (2010). Reactive oxygen species in cancer. Free Radic. Res..

[27] Sgambato A., Zannoni G.F., Faraglia B., Camerini A., Tarquini E., Spada D., Cittadini A. (2004). Decreased expression of the CDK inhibitor p27Kip1 and increased oxidative DNA damage in the multistep process of cervical carcinogenesis. Gynecol. Oncol..

[28] Looi M.L., Dali A.Z.H.M., Ali S.A.M., Ngah W.Z.W., Yusof Y.A.M. (2008). Oxidative damage and antioxidant status in patients with cervical intraepithelial neoplasia and carcinoma of the cervix. Eur. J. Cancer Prev..

[29] Kim S.Y., Kim J.W., Ko Y.S., Koo J.E., Chung H.Y., Lee-Kim Y.C. (2007). Changes in lipid peroxidation and antioxidant trace elements in serum of women with cervical intraepithelial neoplasia and invasive cancer. Nutr. Cancer.

[30] Sun S.Y., Hail N., Lotan R. (2004). Apoptosis as a novel target for cancer chemoprevention. JNCI J. Natl. Cancer Inst..

[31] Numsen H., Cortes M., Drake E.N., Spallholz J.E. (2008). Cancer chemoprevention: A radical perspective. Free Radic. Biol. Med..

[32] Scalbert A., Manach C., Morand C., Rémésy C., Jiménez L. (2005). Dietary polyphenols and the prevention of diseases. Crit. Rev. Food Sci. Nutr..

[33] Lepley D.M., Li B., Birt D.F., Pelling J.C. (1996). The chemopreventive flavonoid apigenin induces G2/M arrest in keratinocytes. Carcinogenesis.

[34] Fresco P., Borges F., Diniz C., Marques M.P.M. (2006). New insights on the anticancer properties of dietary polyphenols. Med. Res. Rev..

[35] Dai J., Mumper R.J. (2010). Plant phenolics: Extraction, analysis and their antioxidant and anticancer properties. Molecules.

[36] D Archivio M., Filesi C., Di Benedetto R., Gargiulo R., Giovannini C., Masella R. (2007). Polyphenols, dietary sources and bioavailability. Ann. Ist. Super. Sanita.

[37] McNaught A.D., Wilkinson A. (1997). Flavonoids (Isoflavonoids and Neoflavonoids). IUPAC Compendium of Chemical Terminology.

[38] Xiao Z.-P., Peng Z.Y., Peng M.J., Yan W.B., Ouyang Y.Z., Zhu H.L. (2011). Flavonoids health benefits and their molecular mechanism. Mini Rev. Med. Chem..

[39] Beecher G.R. (2003). Overview of dietary flavonoids: Nomenclature, occurrence and intake. J. Nutr..

[40] Boyer J., Liu R.H. (2004). Apple phytochemicals and their health benefits. Nutr. J..

[41] Somerset S.M., Johannot L. (2008). Dietary flavonoid sources in Australian adults. Nutr. Cancer.

[42] Zamora-Ros R., Andres-Lacueva C., Lamuela-Raventós R.M., Berenguer T., Jakszyn P., Barricarte A., Ardanas E., Amiano P., Dorronsoro M., Larranaga N. (2010). Estimation of dietary sources and flavonoid intake in a Spanish adult population (EPIC-Spain). J. Am. Diet. Assoc..

[43] Lambert J.D., Hong J., Yang G., Liao J., Yang C.S. (2005). Inhibition of carcinogenesis by polyphenols: Evidence from laboratory investigations. Am. J. Clin. Nutr..

[44] Tabrez S., Priyadarshini M., Urooj M., Shakil S., Ashraf G.M., Khan M.S., Kamal M.A., Alam Q., Jabir N.R., Abuzenadah A.M. (2013). Cancer chemoprevention by polyphenols and their potential application as nanomedicine. J. Environ. Sci. Health.

[45] Khan N., Afaw F., Mukhtar H. (2008). Cancer chemoprevention through dietary antioxidants: Progress and promise. Antioxid. Redox Signal..

[46] Lee K.W., Lee H.J. (2006). The roles of polyphenols in cancer chemoprevention. Biofactors.

[47] Kampa M., Nifli A.-P., Notas G., Castanas E. (2007). Polyphenols and cancer cell growth. Rev. Physiol. Biochem. Pharmacol..

[48] Khan N., Mukhtar H. (2008). Multitargeted therapy of cancer by green tea polyphenols. Cancer Lett..

[49] Yang R.Y., Lin S., Kuo G. (2008). Content and distribution of flavonoids among 91 edible plant species. Asia Pac. J. Clin. Nutr..

[50] Calderon-Montaño J.M., Burgos-Moron E., Perez-Guerrero C., Lopez-Lazaro M. (2011). A review on the dietary flavonoid kaempferol. Mini Rev. Med. Chem..

[51] Aniya Y., Koyama T., Miyagi C., Miyahira M., Inomata C., Kinoshita S., Ichiba T. (2005). Free radical scavenging and hepatoprotective actions of the medicinal herb, Crassocephalum crepidioides from the Okinawa Islands. Biol. Pharm. Bull..

[52] Lehtonen H.M., Lehtinen O., Suomela J.P., Viitanen M., Kallio H. (2010). Flavonol glycosides of sea buckthorn (*Hippophae rhamnoides* ssp. sinensis) and lingonberry (*Vaccinium vitis-idaea*) are bioavailable in humans and monoglucuronidated for excretion. J. Agric. Food Chem..

[53] Bonetti A., Marotti I., Dinelli G. (2007). Urinary excretion of kaempferol from common beans (*Phaseolus vulgaris* L.) in humans. Int. J. Food Sci. Nutr..

[54] Barve A., Chen C., Hebbar V., Desiderio J., Saw C.L., Kong A.N. (2009). Metabolism, oral bioavailability and pharmacokinetics of chemopreventive kaempferol in rats. Biopharm. Drug Dispos..

[55] Radtke J., Linseisen J., Wolfram G. (2002). Fasting plasma concentrations of selected flavonoids as markers of their ordinary dietary intake. Eur. J. Nutr..

[56] Cao J., Zhang Y., Chen W., Zhao X. (2010). The relationship between fasting plasma concentrations of selected flavonoids and their ordinary dietary intake. Br. J. Nutr..

[57] Sanz M.J., Ferrandiz M.L., Cejudo M., Terencio M.C., Gil B., Bustos G., Ubeda A., Gunasegaran R., Alcaraz M.J. (1994). Influence of a series of natural flavonoids on free radical generating systems and oxidative stress. Xenobiotica.

[58] Verma A.R., Vijayakumar M., Mathela C.S., Rao C.V. (2009). In vitro and in vivo antioxidant properties of different fractions of *Moringa oleifera* leaves. Food Chem. Toxicol..

[59] Pietta P.G. (2000). Flavonoids as antioxidants. J. Nat. Prod..

[60] Hibatallah J., Carduner C., Poelman M.C. (1999). In Vivo and in vitro assessment of the free-radical-scavenger activity of Ginkgo flavone glycosides at high concentration. J. Pharm. Pharmacol..

[61] Bonina F., Puglia C., Ventura D., Aquino R., Tortora S., Sacchi A., Saija A., Tomaino A., Pellegrino M.L., de Caprariis P. (2002). In vitro antioxidant and in vivo photoprotective effects of a lyophilized extract of *Capparis spinosa* L buds. J. Cosmet. Sci..

[62] Kampkotter A., Gombitang N.C., Zurawski R.F., Timpel C., Chovolou Y., Watjen W., Kahl R. (2007). Effects of the flavonoids kaempferol and fisetin on thermotolerance, oxidative stress and FoxO transcription factor DAF-16 in the model organism *Caenorhabditis elegans*. Arch. Toxicol..

[63] Russo M., Spagnuolo C., Tedesco I., Bilotto S., Russo G.L. (2012). The flavonoid quercetin in disease prevention and therapy: Facts and fancies. Biochem. Pharmacol..

[64] Graefe E.U., Wittig J., Mueller S., Riethling A.K., Uehleke B., Drewelow B., Pforte H., Jacobasch G., Derendorf H., Veit M. (2001). Pharmacokinetics and bioavailability of quercetin glycosides in humans. J. Clin. Pharmacol..

[65] Olthof M.R., Hollman P.C.H., Vree T.B., Katan M.B. (2000). Bioavailabilities of quercetin-3-glucoside and quercetin-4′-glucoside do not differ in humans. J. Nutr..

[66] Day A.J., Mellon F., Barron D., Sarrazin G., Morgan M.R., Williamson G. (2001). Human metabolism of dietary flavonoids: Identification of plasma metabolites of quercetin. Free Radic. Res..

[67] Sawai Y., Kohsaka K., Nishiyama Y., Ando K. (1987). Serum concentrations of rutoside metabolites after oral administration of a rutoside formulation to humans. Arzneim. Forsch..

[68] Noroozi M., Burns J., Crozier A., Kelly I.E., Lean M.E. (2000). Prediction of dietary flavonol consumption from fasting plasma concentration or urinary excretion. Eur. J. Clin. Nutr..

[69] Erlund I., Silaste M.L., Alfthan G., Rantala M., Kesäniemi Y.A., Aro A. (2002). Plasma concentrations of the flavonoids hesperetin, naringenin and quercetin in human subjects following their habitual diets, and diets high or low in fruit and vegetables. Eur. J. Clin. Nutr..

[70] Harwood M., Danielewska-Nikiel B., Borzelleca J.F., Flamm G.W., Williams G.M., Lines T.C. (2007). A critical review of the data related to the safety of quercetin and lack of evidence of in vivo toxicity, including lack of genotoxic/carcinogenic properties. Food Chem. Toxicol..

[71] Manach C., Williamson G., Morand C., Scalbert A., Rémésy C. (2005). Bioavailability and bioefficacy of polyphenols in humans: I. Review of 97 bioavailability studies. Am. J. Clin. Nutr..

[72] Conquer J.A., Maiani G., Azzini E., Raguzzini A., Holub B.J. (1998). Supplementation with quercetin markedly increases plasma quercetin concentration without effect on selected risk factors for heart disease in healthy subjects. J. Nutr..

[73] Hanahan D., Weinberg R.A. (2011). Hallmarks of cancer: The next generation. Cell.

[74] Boots A.W., Haenen G.R., Bast A. (2008). Health effects of quercetin: From antioxidant to nutraceutical. Eur. J. Pharmacol..

[75] Singh M., Kaur M., Silakari O. (2014). Flavones: An important scaffold for medicinal chemistry. Eur. J. Med. Chem..

[76] Manach C., Scalbert A., Morand C., Remesy C., Jimenez L. (2004). Polyphenols: Food sources and bioavailability. Am. J. Clin. Nutr..

[77] Yang C.S., Landau J.M., Huang M.T., Newmark H.L. (2001). Inhibition of carcinogenesis by dietary polyphenolic compounds. Annu. Rev. Nutr..

[78] Nielsen S.E., Young J.F., Daneshvar B., Lauridsen S.T., Knuthsen P., Sandström B., Dragsted L.O. (1999). Effect of parsley (*Petroselinum crispum*) intake on urinary apigenin excretion, blood antioxidant enzymes and biomarkers for oxidative stress in human subjects. Br. J. Nutr..

[79] Janssen K., Mensink R.P., Cox F.J.J., Harryvan J.L., Hovenier R., Hollman P.C.H., Katan M.B. (1998). Effects of the fl avonoids quercetin and apigenin on hemostasis in healthy volunteers: Results from an in vitro and a dietary supplement study. Am. J. Clin. Nutr..

[80] Chen J., Lin H., Hu M. (2002). Metabolism of flavonoids via enteric recycling: Role of intestinal disposition. Pharmacol. Exp. Ther..

[81] Oh E.K., Kim H.J., Bae S.M., Park M.Y., Kim Y.W., Kim T.E., Ahn W.S. (2008). Apigenin-induced apoptosis in cervical cancer cell lines. Korean J. Obstet. Gynec..

[82] Zheng P.W., Chiang L.C., Lin C.C. (2005). Apigenin induced apoptosis through p53-dependent pathway in human cervical carcinoma cells. Life Sci..

[83] Czyz J., Madeja Z., Irmer U., Korohoda W., Hulser D.F. (2005). Flavonoid apigenin inhibits motility and invasiveness of carcinoma cells in vitro. Int. J. Cancer.

[84] Setchell K.D., Brown N.M., Zimmer-Nechemias L., Brashear W.T., Wolfe B.E., Kirschner A.S., Heubi J.E. (2002). Evidence for lack of absorption of soy isoflavone glycosides in humans, supporting the crucial role of intestinal metabolism for bioavailability. Am. J. Clin. Nutr..

[85] Izumi T., Piskula M.K., Osawa S., Obata A., Tobe K., Saito M., Kataoka S., Kikuchi M. (2000). Soy isoflavone aglycones are absorbed faster and in higher amounts than their glucosides in humans. J. Nutr..

[86] Day A.J., DuPont M.S., Ridley S., Rhodes M., Rhodes M.J., Morgan M.R. (1998). Deglycosylation of flavonoid and isoflavonoid glycosides by human small intestine and liver β-glucosidase activity. FEBS Lett..

[87] Joannou G.E., Kelly G.E., Reeder A.Y., Waring M., Nelson C. (1995). A urinary profile study of dietary phytoestrogens. The identification and mode of metabolism of new isoflavonoids. J. Steroid Biochem. Mol. Biol..

[88] Adlercreutz H., Honjo H., Higashi A., Fotsis T., Hamalainen E., Hasegawa T., Okada H. (1991). Urinary excretion of lignans and isoflavonoid phytoestrogens in Japanese men and women consuming a traditional Japanese diet. Am. J. Clin. Nutr..

[89] Watanabe S., Yamaguchi M., Sobue T., Takahashi T., Miura T., Arai Y., Mazur W., Wahala K., Adlercreutz H. (1998). Pharmacokinetics of soybean isoflavones in plasma, urine, and feces of men after ingestion of 60 g baked soybean powder (Kinako). J. Nutr..

[90] Yashar C.M., Spanos W.J., Taylor D.D., Gercel-Taylor C. (2005). Potentiation of the radiation effect with genistein in cervical cancer cells. Gynecol. Oncol..

[91] Hillman G.G., Forman J.D., Kucuk O., Yudelev M., Maughan R.L., Rubio J., Sarkar F.H. (2001). Genistein potentiates the radiation effect on prostate carcinoma cells. Clin. Cancer Res..

[92] Akimoto T., Nonaka T., Ishikawa H., Sakurai H., Saitoh J.I., Takahashi T., Mitsuhashi N. (2001). Genistein, a tyrosine kinase inhibitor, enhanced radiosensitivity in human esophageal cancer cell lines in vitro: Possible involvement of inhibition of survival signal transduction pathways. Int. J. Radiat. Oncol. Biol. Phys..

[93] Akiyama T., Ogawara H. (1991). Use and specificity of genistein as inhibitor of protein-tyrosine kinases. Methods Enzymol..

[94] Markovits J., Larsen A.K., Segal-Bendirdjian E., Fosse P., Sancier T.M., Gazit A., Levitzki A., Umezawa K., Jacquemin-Sablon A. (1994). Inhibition of DNA topoisomerases I and II and induction of apoptosis by erbstatin and tyrphostin derivatives. Biochem. Pharmacol..

[95] Cassidy A., Bingham S., Setchell K.D. (1994). Biological effect of a diet of soy protein rich in isoflavones on the menstrual cycle of premenopausal women. Am. J. Clin. Nutr..

[96] Wei H., Wei L., Frenkel K., Bowen R., Barnes S. (1993). Inhibition of tumorpromoter-induced hydrogen peroxide formation in vitro and in vivo by genistein. Nutr. Cancer.

[97] Yanagihara K., Ito A., Toge T., Numoto M. (1993). Antiproliferative effect of isoflavones on human cancer cell lines established from the gastrointestinal tract. Cancer Res..

[98] Busby M.G., Jeffcoat A.R., Bloedon M.A., Koch M.A., Black T., Dix K.J., Heizer K., Thomas B., Hill J., Crowell J. (2002). Clinical characteristics and pharmacokinetics of purified soy isoflavones: Single-dose administration to healthy men. Am. J. Clin. Nutr..

[99] Zhang B., Liu J.Y., Pan J.S., Han S.P., Yin X.X., Wang B., Hu G. (2006). Combined treatment of ionizing radiation with genistein on cervical cancer HeLa cells. J. Pharmacol. Sci..

[100] Guo J.M., Kang G.Z., Xiao B.X., Liu D.H., Zhang S. (2004). Effect of daidzein on cell growth, cell cycle, and telomerase activity of human cervical cancer in vitro. Int. J. Gynecol. Cancer.

[101] Huber G.M., Rupasinghe H.P.V. (2009). Phenolic profiles and antioxidant properties of apple skin extracts. J. Food Sci..

[102] Ratnasooriya C., Rupasinghe H.P.V., Jamieson A. (2010). Juice quality and polyphenol concentration of fresh fruits and pomace of selected Nova Scotia-grown grape cultivars. Can. J. Plant Sci..

[103] Otaki N., Kimira M., Katsumata S., Uehara M., Watanabe S., Suzuki K. (2009). Distribution and major sources of flavonoid intakes in the middle-aged Japanese women. J. Clin. Biochem. Nutr..

[104] Ullmann U., Haller J., Decourt J.P., Girault N., Girault J., Richard-Caudron A.S., Pineau B., Weber P. (2003). A single ascending dose study of epigallocatechin gallate in healthy volunteers. J. Int. Med. Res..

[105] Van Amelsvoort J.M., Van Hof K.H., Mathot J.N., Mulder T.P., Wiersma A., Tijburg L.B. (2001). Plasma concentrations of individual tea catechins after a single oral dose in humans. Xenobiotica.

[106] Natsume M., Osakabe N., Oyama M., Sasaki M., Baba S., Nakamura Y., Osawa T., Terao J. (2003). Structures of (−)-epicatechin glucuronide identified from plasma and urine after oral ingestion of (−)-epicatechin: Differences between human and rat. Free Radic. Biol. Med..

[107] Meng X., Sang S., Zhu N., Lu H., Sheng S., Lee M.-J., Ho C.-T., Yang C.S. (2002). Identification and characterization of methylated and ring-fission metabolites of tea catechins formed in humans, mice, and rats. Chem. Res. Toxicol..

[108] Lee M.J., Maliakal P., Chen L., Meng X., Bondoc F.Y., Prabhu S., Lambert G., Mohr S., Yang C.S. (2002). Pharmacokinetics of tea catechins after ingestion of green tea and (−)-epigallocatechin-3-gallate by humans: Formation of different metabolites and individual variability. Cancer Epidemiol. Biomark. Prev..

[109] Chow H.H.S., Cai Y., Alberts D.S., Hakim I., Dorr R., Shahi F., Crowell J.A., Yang C.S., Hara Y. (2001). Phase I pharmacokinetic study of tea polyphenols following single-dose administration of epigallocatechin gallate and polyphenon E. Cancer Epidemiol. Biomark. Prev..

[110] Meng X., Lee M.J., Li C., Sheng S., Zhu N., Sang S., Ho C.-T., Yang C.S. (2001). Formation and identification of 4′-*O*-methyl-(−)-epigallocatechin in humans. Drug Metab. Dispos..

[111] Ahn W.S., Huh S.W., Bae S.M., Lee I.P., Lee J.M., Namkoong S.E., Kim C.K., Sin J.-I. (2003). A major constituent of green tea, EGCG, inhibits the growth of a human cervical cancer cell line, CaSki cells, through apoptosis, G1 arrest, and regulation of gene expression. DNA Cell Biol..

[112] Hause Z. (2002). Papilomaviruses and cancer: From basic studies to clinical application. Nat. Rev. Cancer.

[113] Moody C.A., Laiminis L.A. (2010). Human papillomavirus oncoproteins: Pathways to transformation. Nat. Rev. Cancer.

[114] Von Knebel Doeberitz M. (2002). New markers for cervical dysplasia to visualize the genomic chaos created by aberrant oncogenic papillomavirus infection. Eur. J. Cancer.

[115] Qiao Y., Cao J., Xie L., Shi X. (2009). Cell growth inhibition and gene expression regulation by (−)-epigallocatechin-3-gallate in human cervical cancer cells. Arch. Pharm. Res..

[116] Sharma C., Nusri Q.E.A., Begum S., Javed E., Rizvi T.A., Hussain A. (2012). Epigallocatechin-3-gallate induces apoptosis and inhibits invasion and migration of human cervical cancer cells. Asian Pac. J. Cancer Prev..

[117] Zou C., Liu H., Feugang J.M., Hao Z., Chow H.S., Garcia F. (2010). Green tea compound in chemoprevention of cervical cancer. Int. J. Gynecol. Cancer.

[118] Tripoli E., Guardia M.L., Giammanco S., Danila D., Majo D., Giammanco M. (2007). Citrus flavonoids: Molecular structure, biological activity and nutritional properties: A review. Food Chem..

[119] Manthey J.A., Grohmann K., Guthrie N. (2001). Biological properties of citrus flavonoids pertaining to cancer and inflammation. Curr. Med. Chem..

[120] Amaro M.I., Rocha J., Vila-Real H., Eduardo-Figueira M., Mota-Filipe H., Sepodes B., Ribeiro M.H. (2009). Anti-inflammatory activity of naringin and the biosynthesised naringenin by naringinase immobilized in microstructured materials in a model of DSS-induced colitis in mice. Food Res. Int..

[121] Choi J.S., Park K.Y., Moon S.H., Rhee S.H., Young H.S. (1994). Antimutagenic effect of plant flavonoids in the salmonella assay system. Arch. Pharm. Res..

[122] Kanno S., Tomizawa A., Ohtake T., Koiwai K., Ujibe M., Ishikawa M. (2006). Naringenininduced apoptosis via activation of NF-κB and necrosis involving the loss of ATP in human promyeloleukemia HL-60 cells. Toxicol. Lett..

[123] Wang B.D., Yang Z.Y., Wang Q., Cai T.K., Crewdson P. (2006). Synthesis, characterization, cytotoxic activities and DNA-binding properties of the La(III) complex with naringenin schiff-base. Bioorg. Med. Chem..

[124] Ratnam D.V., Ankola D.D., Baradwaj V., Sahana D.K., Ravikumar M.N.V. (2006). Role of antioxidants in prophylaxis and therapy: A pharmaceutical perspective. J. Control. Release.

[125] Krishnakumar N., Sulfikkarali N., RajendraPrasad N., Karthikeyan S. (2011). Enhanced anticancer activity of naringenin-loaded nanoparticles in human cervical (HeLa) cancer cells. Biomed. Prev. Nutr..

[126] Yen F.L., Wu T.H., Lin L.T., Cham T.M., Lin C.C. (2008). Naringenin-loaded nanoparticles improve the physicochemical properties and the hepatoprotective effect of naringenin in orally administrated rats with CCl_4_-induced acute liver failure. Pharm. Res..

[127] Maeda H., Wu J., Sawa T., Matsumura Y., Flori K. (2000). Tumor vascular permeability and the EPR effect in macromolecular therapeutics: A review. J. Control. Release.

[128] Park J.W., Lee J.W., Paik H.D., Cho S.G., Nah S.Y., Park Y.S., Han Y.S. (2010). Cytotoxic effect of 7-Obutyl naringenin on human breast cancer MCF-7 cells. Food Sci. Biotechnol..

[129] Chang H., Mi M., Ling W., Zhu J., Zhang Q., Wei N., Zhou Y., Tang Y., Yuan J. (2008). Structurally related cytotoxic effect of flavonoids on human cancer cells in vitro. Arch. Pharm. Res..

[130] Ramesh E., Alshatwi A.A. (2013). Naringin induces death receptor and mitochondria-mediated apoptosis in human cervical cancer (SiHa) cells. Food Chem. Toxicol..

[131] Zeng L., Zhen Y., Chen Y., Zou L., Zhang Y., Hu F., Feng J., Shen J., Wei B. (2014). Naringin inhibits growth and induces apoptosis by a mechanism dependent on reduced activation of NF-κB/COX-2-caspase-1 pathway in HeLa cervical cancer cells. Int. J. Oncol..

[132] Vanamala J., Leonardi T., Patil B.S., Taddeo S.S., Murphy M.E., Pike L.M., Chapkin R.S., Lupton J.R., Turner N.D. (2006). Suppression of colon carcinogenesis by bioactive compounds in grapefruit. Carcinogenesis.

[133] Kim D.-I., Lee S.J., Lee S.B., Park K., Kim W.J., Moon S.K. (2008). Requirement for Ras/Raf/ERK pathway in naringin-induced G1-cell-cycle arrest via p21WAF1 expression. Carcinogenesis.

[134] Kahkonen M.P., Heinonen M. (2003). Antioxidant activity of anthocyanins and their aglycons. J. Agric. Food Chem..

[135] Kuhnau J. (1976). The flavonoids. A class of semi-essential food components: Their role in human nutrition. World Rev. Nutr. Diet..

[136] Cevallos-Casals B.A., Byrne D., Okie W.R., Cisneros-Zevallos L. (2006). Selecting new peach and plum genotypes rich in phenolic compounds and enhanced functional properties. Food Chem..

[137] Chen P.N., Kuo W.H., Chiang C.L., Chiou H.L., Hsieh Y.S., Chu S.C. (2006). Black rice anthocyanins inhibit cancer cells invasion via repressions of MMPs and u-PA expression. Chem. Biol. Interact..

[138] Middleton E., Kandaswami C., Theoharides T.C. (2000). The effects of plant flavonoids on mammalian cells: Implications for inflammation, heart disease, and cancer. Pharmacol. Rev..

[139] Jang M., Cai L., Udeani G.O., Slowing K.V., Thomas C.F., Beecher C.W., Fong H., Farnsworth R., Kinghorn A.D., Mehta R.G. (1997). Cancer chemopreventive activity of resveratrol, a natural product derived from grapes. Science.

[140] Chen C.C., Hsu J.D., Wang S.F., Chiang H.C., Yang M.Y., Kao E.S., Ho Y.-C., Wang C.J. (2003). Hibiscus sabdariffa extract inhibits the development of atherosclerosis in cholesterol-fed rabbits. J. Agric. Food Chem..

[141] Seeram N.P., Zhang Y., Nair M.G. (2003). Inhibition of proliferation of human cancer cells and cyclooxygenase enzymes by anthocyanidins and catechins. Nutr. Cancer.

[142] Fotsis T., Pepper M.S., Aktas E., Breit S., Rasku S., Adlercreutz H., Wahala K., Montesano R., Schweigerer L. (1997). Flavonoids, dietary-derived inhibitors of cell proliferation and in vitro angiogenesis. Cancer Res..

[143] Song Q.I.A.N., Li-qin J.I.N. (2008). The studies of cyanidin 3-glucoside-induced apoptosis in human cervical cancer Hela cells and its mechanism. Chin. J. Biochem. Pharm..

[144] Shahidi F., Naczk M. (1995). Food Phenolics, Sources, Chemistry, Effects, Applications.

[145] Faried A., Kurnia D., Faried L.S., Usman N., Miyazaki T., Kato H., Kuwano H. (2007). Anticancer effects of gallic acid isolated from Indonesian herbal medicine. *Phaleria macrocarpa* (Scheff.) Boerl, on human cancer cell lines. Int. J. Oncol..

[146] You B.R., Moon H.J., Han Y.H., Park W.H. (2010). Gallic acid inhibits the growth of HeLa cervical cancer cells via apoptosis and/or necrosis. Food Chem. Toxicol..

[147] Clifford M.N., Scalbert A. (2000). Ellagitannins—Occurrence in food, bioavailability and cancer prevention. J. Food Sci. Agric..

[148] Olthof M.R., Hollman P.C.H., Katan M.B. (2001). Chlorogenic acid and caffeic acid are absorbed in humans. J. Nutr..

[149] Sosulski F., Krygier K., Hogge L. (1982). Free, esterified, and insoluble-bound phenolic acids. 3. Composition of phenolic acids in cereal and potato flours. J. Agric. Food Chem..

[150] Van de Putte B., Hollman P.C.H. (2000). Catechin contents of foods commonly consumed in The Netherlands. 1. Fruits, vegetables, staple foods, and processed foods. J. Agric. Food Chem..

[151] Kern S.M., Bennett R.N., Mellon F.A., Kroon P.A., Garcia-Conesa M.T. (2003). Absorption of hydroxycinnamates in humans after high-bran cereal consumption. J. Agric. Food Chem..

[152] Sharma R.A., Gescher A.J., Steward W.P. (2005). Curcumin: The story so far. Eur. J. Cancer.

[153] Anand P., Kunnumakkara A.B., Newman R.A., Aggarwal B.B. (2007). Bioavailability of curcumin: Problems and promises. Mol. Pharm..

[154] Bar-Sela G., Epelbaum R., Schaffer M. (2010). Curcumin as an anti-cancer agent: Review of the gap between basic and clinical applications. Curr. Med. Chem..

[155] Saengkrit N., Saesoo S., Srinuanchai W., Phunpee S., Ruktanonchai U.R. (2014). Influence of curcumin-loaded cationic liposome on anticancer activity for cervical cancer therapy. Colloids Surf. B Biointerfaces.

[156] Wahlström B., Blennow G. (1978). A study on the fate of curcumin in the rat. Acta Pharmacol. Toxicol..

[157] Shoba G., Joy D., Joseph T., Majeed M., Rajendran R., Srinivas P.S. (1998). Influence of piperine on the pharmacokinetics of curcumin in animals and human volunteers. Planta Med..

[158] Ryu E.K., Choe Y.S., Lee K.H., Choi Y., Kim B.T. (2006). Curcumin and dehydrozingerone derivatives: Synthesis, radiolabeling, and evaluation for β-amyloid plaque imaging. J. Med. Chem..

[159] Prasad S., Tyagi A.K., Aggarwal B.B. (2014). Recent developments in delivery, bioavailability, absorption and metabolism of curcumin: The golden pigment from golden spice. Cancer Res. Treat..

[160] Aggarwal B.B., Bhatt I.D., Ichikawa H., Ahn K.S., Sethi G., Sandur S.K., Natarajan C., Seeram N., Shishodia S., Ravindran P.N., Babu K.N., Sivaraman K. (2007). Curcumin—Biological and medicinal properties. Turmeric the Genus Curcuma.

[161] Limtrakul P., Chearwae W., Shukla S., Phisalphong C., Ambudkar S.V. (2007). Modulation of function of three ABC drug transporters, P-glycoprotein (ABCB1), mitoxantrone resistance protein (ABCG2) and multidrug resistance protein 1 (ABCC1) by tetrahydrocurcumin, a major metabolite of curcumin. Mol. Cell. Biochem..

[162] Goel A., Kunnumakkara A.B., Aggarwal B.B. (2007). Curcumin as Curecumin: From kitchen to clinic. Biochem. Pharmacol..

[163] Shahrzad S., Aoyagi K., Winter A., Koyama A., Bitsch I. (2001). Pharmacokinetics of gallic acid and its relative bioavailability from tea in healthy humans. J. Nutr..

[164] Seeram N.P., Lee R., Heber D. (2004). Bioavailability of ellagic acid in human plasma after consumption of ellagitannins from pomegranate (Punica granatum L.) juice. Clin. Chim. Acta.

[165] Strimpakos A.S., Sharma R.A. (2008). Curcumin: Preventive and therapeutic properties in laboratory studies and clinical trials. Antioxid. Redox Signal..

[166] Hatcher H., Planalp R., Cho J., Torti F.M., Torti S.V. (2008). Curcumin: From ancient medicine to current clinical trials. Cell. Mol. Life Sci..

[167] Goel A., Jhurani S., Aggarwal B.B. (2008). Multi-targeted therapy by curcumin: How spicy is it?. Mol. Nutr. Food Res..

[168] Maheshwari R., Singh A.K., Gaddipati J., Srimal R.C. (2006). Multiple biological activities of curcumin: A short review. Life Sci..

[169] Aggarwal B.B., Harikumar K.B. (2009). Potential therapeutic effects of curcumin, the anti-inflammatory agent, against neurodegenerative, cardiovascular, pulmonary, metabolic, autoimmune and neoplastic diseases. Int. J. Biochem. Cell Biol..

[170] Prusty B.K., Das B.C. (2005). Constitutive activation of transcription factor AP-1 in cervical cancer and suppression of human papillomavirus (HPV) transcription and AP-1 activity in HeLa cells by curcumin. Int. J. Cancer.

[171] Berezutskaya E., Bagchi S. (1997). The human papillomavirus E7 oncoprotein functionally interacts with the S4 subunit of the 26 S proteasome. J. Biol. Chem..

[172] Smitha B., Sreekanth C.N., Thulasidasan A.K.T., Anto N.P., Cheriyan V.T., Puliyappadamba V.T., Menon S.G., Ravichandran S.D., Anto R.J. (2011). Akt is upstream and MAPKs are downstream of NF-κB in paclitaxel-induced survival signaling events, which are down-regulated by curcumin contributing to their synergism. Int. J. Biochem. Cell Biol..

[173] Ganta S., Mansoor A. (2009). Coadministration of paclitaxel and curcumin in nanoemulsion formulations to overcome multidrug resistance in tumor cells. Mol. Pharm..

[174] Cheng A.L., Hsu C.H., Lin J.K., Hsu M.M., Ho Y.F., Shen T.S., Ko J.Y., Lin J.-T., Lin B.-R., Wu M.S. (2001). Phase I clinical trial of curcumin, a chemopreventive agent, in patients with high-risk or pre-malignant lesions. Anticancer Res..

[175] Kunwar A., Sandur S.K., Krishna M., Priyadarsini K.I. (2009). Curcumin mediates time and concentration dependent regulation of redox homeostasis leading to cytotoxicity in macrophage cells. Eur. J. Pharmacol..

[176] Fuchs J.R., Pandit B., Bhasin D., Etter J.P., Regan N., Abdelhamid D., Li C., Lin H., Li P.K. (2009). Structure-activity relationship studies of curcumin analogues. Bioorg. Med. Chem. Lett..

[177] Maher D.M., Bell M.C., O’Donnell E.A., Gupta B.K., Jaggi M., Chauhan S.C. (2011). Curcumin suppresses human papillomavirus oncoproteins, restores p53, rb, and ptpn13 proteins and inhibits benzo[*a*]pyrene-induced upregulation of HPV E7. Mol. Carcinog..

[178] Aggarwal B.B., Bhardwaj A., Aggarwal R.S., Seeram N.P., Shishodia S., Takada Y. (2004). Role of resveratrol in prevention and therapy of cancer: Preclinical and clinical studies. Anticancer Res..

[179] Goldberg D.M., Yan J., Soleas G.J. (2003). Absorption of three wine-related polyphenols in three different matrices by healthy subjects. Clin. Biochem..

[180] Walle T., Hsieh F., DeLegge M.H., Oatis J.E., Walle U.K. (2004). High absorption but very low bioavailability of oral resveratrol in humans. Drug Metab. Dispos..

[181] Lu Z., Zhang Y., Liu H., Yuan J., Zheng Z., Zou G. (2007). Transport of a cancer chemopreventive polyphenol, resveratrol: Interaction with serum albumin and hemoglobin. J. Fluoresc..

[182] Sale S., Verschoyle R.D., Boocock D., Jones D.J.L., Wilsher N., Ruparelia K.C., Potter P.A., Farmer P.B., Steward W.P., Gescher A.J. (2004). Pharmacokinetics in mice and growth-inhibitory properties of the putative cancer chemopreventive agent resveratrol and the synthetic analogue *trans* 3,4,5,4′-tetramethoxystilbene. Br. J. Cancer.

[183] Hsu K.-F., Wu C.L., Huang S.C., Wu C.M., Hsiao J.R., Yo Y.T., Chen Y.H., Shiau A.-L., Chou C.Y. (2009). Cathepsin L mediates resveratrol-induced autophagy and apoptotic cell death in cervical cancer cells. Autophagy.

[184] Kim Y.S., Sull J.W., Sung H.J. (2012). Suppressing effect of resveratrol on the migration and invasion of human metastatic lung and cervical cancer cells. Mol. Boil. Rep..

[185] Zoberi I., Bradbury C.M., Curry H.A., Bisht K.S., Goswami P.C., Roti J.L.R., Gius D. (2002). Radiosensitizing and anti-proliferative effects of resveratrol in two human cervical tumor cell lines. Cancer Lett..

[186] García-Zepeda S.P., García-Villa E., Díaz-Chávez J., Hernández-Pando R., Gariglio P. (2013). Resveratrol induces cell death in cervical cancer cells through apoptosis and autophagy. Eur. J. Cancer Care.

[187] Kramer M.P., Węsierska-Gądek J. (2009). Monitoring of Long-Term Effects of Resveratrol on Cell Cycle Progression of Human HeLa Cells after Administration of a Single Dose. Ann. N. Y. Acad. Sci..

[188] Talwar G.P. (2008). A Clinical Study on the Clearance of Human Papilloma Virus (HPV) Infection in Uterine Cervix by Basant (a Polyherbal Cream) and Curcumin Soft Gelatin Capsule in Females Infected with HPV Clinical Trial Registry—India National Institute of Medical Statistics.

[189] Basu P., Dutta S., Begum R., Mittal S., Dutta P.D., Bharti A.C., Panda K.C., Biswas J., Dey B., Talwar G.P. (2013). Clearance of cervical human papillomavirus infection by topical application of curcumin and curcumin containing polyherbal cream: A phase II randomized controlled study. Asian Pac. J. Cancer Prev..

[190] Ahn W.S., Yoo J., Huh S.W., Kim C.K., Lee J.M., Namkoong S.E., Bae S.-M., Lee I.P. (2003). Protective effects of green tea extracts (polyphenon E and EGCG) on human cervical lesions. Eur. J. Cancer Prev..

[191] Wang S.-J., Zheng C.J., Peng C., Zhang H., Jiang Y.P., Han T., Qin L.P. (2013). Plants and cervical cancer: An overview. Expert Opin. Investig. Drugs.

[192] Priyadarsini R.V., Murugan R.S., Maitreyi S., Ramalingam K., Karunagaran D., Nagini S. (2010). The flavonoid quercetin induces cell cycle arrest and mitochondria-mediated apoptosis in human cervical cancer HeLa cells through p53 induction and NF-κB inhibition. Eur. J. Pharmacol..

[193] Xu W., Liu J., Li C., Wu H.Z., Liu Y.W. (2008). Kaempferol-7-*O*-β-d-glucoside (KG) isolated from *Smilax china* L. rhizome induces G 2/M phase arrest and apoptosis on HeLa cells in a p53-independent manner. Cancer Lett..

[194] Ying T.H., Yang S.F., Tsai S.J., Hsieh S.C., Huang Y.C., Bau D.T., Hsieh Y.H. (2012). Fisetin induces apoptosis in human cervical cancer HeLa cells through ERK1/2-mediated activation of caspase-8-/caspase-3-dependent pathway. Arch. Toxicol..

[195] Yan J., Wang Q., Zheng X., Sun H., Zhou Y., Li D., Lin Y. (2012). Luteolin enhances TNF-related apoptosis-inducing ligand’s anticancer activity in a lung cancer xenograft mouse model. Biochem. Biophys. Res. Commun..

[196] Alshatwi A.A., Ramesh E., Periasamy V.S., Subash-Babu P. (2013). The apoptotic effect of hesperetin on human cervical cancer cells is mediated through cell cycle arrest, death receptor, and mitochondrial pathways. Fundam. Clin. Pharmacol..

[197] Roohbakhsh A., Parhiz H., Soltani F., Rezaee R., Iranshahi M. (2015). Molecular mechanisms behind the biological effects of hesperidin and hesperetin for the prevention of cancer and cardiovascular diseases. Life Sci..

[198] Yokoyama M., Noguchi M., Nakao Y., Ysunaga M., Yamasaki F., Iwasaka T. (2008). Antiproliferative effects of the major tea polyphenol, (−)-epigallocatechin gallate and retinoic acid in cervical adenocarcinoma. Gynecol. Oncol..

[199] Shen X., Shulan L., Zhang J., Li S., Gao J., Pan C. (2009). Effects of Res on proliferation and apoptosis of human cervical carcinoma cell lines C33A, SiHa and HeLa. J. Med. Coll. PLA.

[200] Srinivas G., Anto R.J., Srinivas P., Vidhyalakshmi S., Senan V.P., Karunagaran D. (2003). Emodin induces apoptosis of human cervical cancer cells through poly (ADP-ribose) polymerase cleavage and activation of caspase-9. Eur. J. Pharmacol..

[201] Singh M., Singh N. (2009). Molecular mechanism of curcumin induced cytotoxicity in human cervical carcinoma cells. Mol. Cell. Biochem..

[202] Giridharan P., Somasundaram S.T., Perumal K., Vishwakarma R.A., Karthikeyan N.P., Velmurugan R., Balakrishnan A. (2002). Novel substituted methylenedioxy lignan suppresses proliferation of cancer cells by inhibiting telomerase and activation of c-myc and caspases leading to apoptosis. Br. J. Cancer.

[203] Singh M., Bhui K., Singh R., Shukla Y. (2013). Tea polyphenols enhance cisplatin chemosensitivity in cervical cancer cells via induction of apoptosis. Life Sci..

[204] Di Domenico F., Foppoli C., Coccia R., Perluigi M. (2012). Antioxidants in cervical cancer: Chemopreventive and chemotherapeutic effects of polyphenols. Biochim. Biophys. Acta.

[205] He F., Wang Q., Zheng X.L., Yan J.Q., Yang L., Sun H., Hu N., Lin Y., Wang X. (2012). Wogonin potentiates cisplatin-induced cancer cell apoptosis through accumulation of intracellular reactive oxygen species. Oncol. Rep..

[206] Jakubowicz-Gil J., Paduch R., Piersiak T., Głowniak K., Gawron A., Kandefer-Szerszeń M. (2005). The effect of quercetin of pro-apoptotic activity of cisplatin in HeLa cells. Biochem. Pharmacol..

[207] Xu Y., Xin Y., Diao Y., Lu C., Fu J., Luo L., Yin Z. (2011). Synergistic effects of apigenin and paclitaxel on apoptosis of cancer cells. PLoS ONE.

[208] Lo Y.L., Wang W. (2013). Formononetin potentiates epirubicininduced apoptosis via ROS production in HeLa cells in vitro. Chem. Biol. Interact..

[209] Lo Y.L., Wang W., Ho C.T. (2012). 7,3′,4′-Trihydroxyisoflavone modulates multidrug resistance transporters and induces apoptosis via production of reactive oxygen species. Toxicology.

[210] Lin C., Yu Y., Zhao H.G., Yang A., Yan H., Cui Y. (2012). Combination of quercetin with radiotherapy enhances tumor radiosensitivity in vitro and in vivo. Radiother. Oncol..

[211] Shin J.I., Shim J.H., Kim K.H., Choi H.S., Kim J.W., Lee H.G., Kim B.Y., Park S.N., Park O.J., Yoon D.Y. (2008). Sensitization of the apoptotic effect of gamma-irradiation in genistein-pretreated CaSki cervical cancer cells. J. Microbiol. Biotechnol..

